# Multi-regional alterations in glucose and purine metabolic pathways in the Parkinson’s disease dementia brain

**DOI:** 10.1038/s41531-023-00488-y

**Published:** 2023-04-20

**Authors:** Melissa Scholefield, Stephanie J. Church, George Taylor, David Knight, Richard D. Unwin, Garth J. S. Cooper

**Affiliations:** 1 Division of Cardiovascular Sciences, School of Medical Sciences, Faculty of Biology, Medicine and Health, The University of Manchester, Manchester Academic Health Science Centre, Manchester, M13 9NT UK; 2 Biological Mass Spectrometry Core Research Facility, Faculty of Biology, Medicine and Health, The University of Manchester, Manchester, M13 9PT UK; 3 Stoller Biomarker Discovery Centre & Division of Cancer Sciences, School of Medical Sciences, Faculty of Biology, Medicine and Health, The University of Manchester, Citylabs 1.0 (Third Floor), Nelson Street, Manchester, M13 9NQ UK; 4 School of Biological Sciences, Faculty of Science, University of Auckland, Private Bag 92 019, Auckland, 1142 New Zealand

**Keywords:** Cognitive ageing, Dementia, Parkinson's disease, Parkinson's disease, Parkinson's disease

## Abstract

Parkinson’s disease (PD) is one of the most common neurodegenerative diseases, most commonly characterised by motor dysfunction, but also with a high prevalence of cognitive decline in the decades following diagnosis—a condition known as Parkinson’s disease dementia (PDD). Although several metabolic disruptions have been identified in PD, there has yet to be a multi-regional analysis of multiple metabolites conducted in PDD brains. This discovery study attempts to address this gap in knowledge. A semi-targeted liquid chromatography–mass spectrometry analysis of nine neuropathologically-confirmed PDD cases vs nine controls was performed, looking at nine different brain regions, including the cingulate gyrus, cerebellum, hippocampus, motor cortex, medulla, middle temporal gyrus, pons, substantia nigra and primary visual cortex. Case–control differences were determined by multiple t-tests followed by 10% FDR correction. Of 64 identified analytes, 49 were found to be altered in at least one region of the PDD brain. These included metabolites from several pathways, including glucose and purine metabolism and the TCA cycle, with widespread increases in fructose, inosine and ribose-5-phosphate, as well as decreases in proline, serine and deoxyguanosine. Higher numbers of alterations were observed in PDD brain regions that are affected during earlier α-synuclein Braak stages—with the exception of the cerebellum, which showed an unexpectedly high number of metabolic changes. PDD brains show multi-regional alterations in glucose and purine metabolic pathways that reflect the progression of α-synuclein Braak staging. Unexpectedly, the cerebellum also shows a high number of metabolic changes.

## Background

Parkinson’s disease (PD) is one of the most common neurodegenerative diseases, affecting 1% of people over the age of 80 in the UK alone, and with cases increasing rapidly—with the potential for as much as a doubling of cases numbers by 2040 in comparison to 2015 in the United States^1,2^. PD primarily manifests as motor dysfunction, with the main symptoms including resting tremor, bradykinesia and rigidity. However, a significant proportion of those suffering from PD will also go on to develop concurrent cognitive decline, a condition known as Parkinson’s disease dementia (PDD); this can affect up to 80% of PD sufferers by 20 years post-initial diagnosis^3^.

Although PD/PDD are primarily characterised neuropathologically by widespread dopaminergic neuronal loss and Lewy body deposition, with the substantia nigra (SN) pars compacta being the most severely affected region of the brain, these conditions have also previously been observed to show several metabolic dysregulations—both within and outside the SN; current metabolomic perturbations reported in the PD/PDD brain include alterations in the glucose^4–6^, purine metabolism^7^ and tricarboxylic acid (TCA) cycle metabolic pathways^8^. These findings suggest deficiencies in mitochondrial function, increased oxidative stress, and decreased energy production—all of which have been observed in PD/PDD and may contribute to the pathogenesis of the disease^9–12^.

However, studies of brain tissue metabolomics in PD/PDD have thus far been limited and are usually restricted to cover only the basal ganglia or cortex^4–7,13,14^ and/or with no distinction made between PD with or without dementia^15^. A multi-regional, semi-targeted metabolomic analysis of the brain has yet to be carried out in PD/PDD. The aim of this study was to perform such an analysis on confirmed PDD post-mortem brain tissues, firstly in order to investigate PDD case–control differences in cerebral metabolite levels, and, secondly, to observe how any such changes are distributed across brain regions.

In order to do this, a high-performance liquid chromatography–mass spectrometry (HPLC–MS) semi-targeted metabolomics method was developed that would allow us to investigate multiple metabolites across several regions of the PDD brain. These findings could then be used to build a picture of metabolic dysfunction as it occurs in PDD.

## Results

### Cohort characteristics

Details of individual donor samples can be found in Supplementary Material A and Table 1, with a summary shown in Supplementary Material A and Table 2. Cases and controls were matched by age, sex, brain weight and post-mortem delay (PMD) in all regions except the substantia nigra (SN), in which PMD was slightly lower in cases (mean 14.6 h) compared to controls (mean 20.6 h; *p* = 0.03).

### HPLC–MS analysis

**Fig. 1:**
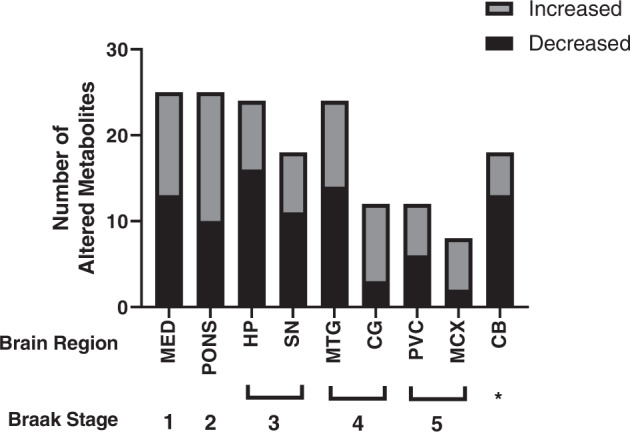
Number of altered metabolites in PDD brain regions. CB cerebellum, CG cingulate gyrus, HP hippocampus, MCX motor cortex, MED medulla oblongata, MTG middle temporal gyrus, PVC primary visual cortex, SN substantia nigra. *CB is typically considered unaffected in α-synuclein Braak staging.

A total of 64 metabolites were identified in the samples (see Supplementary Material 2 for the full list). Of these, 49 (77%) showed significant case–control changes (*p* < 0.05, corrected with 10% FDR) in at least one investigated brain region (see Supplementary Material 1 and Fig. 1 for graphs of all altered metabolites). A total of 27 (57%) of these altered metabolites were decreased in PDD cases compared to controls in at least one region and 21 (45%) showed increases in at least one region. For some analytes, results had to be excluded due to high-blank values (see Table 2). A representative heat map displaying relative case–control fold changes of all identified metabolites in the hippocampus (HP) is included in Fig. 1, with heat maps of other regions included in Additional File 1 and Fig. 3.

**Fig. 2:**
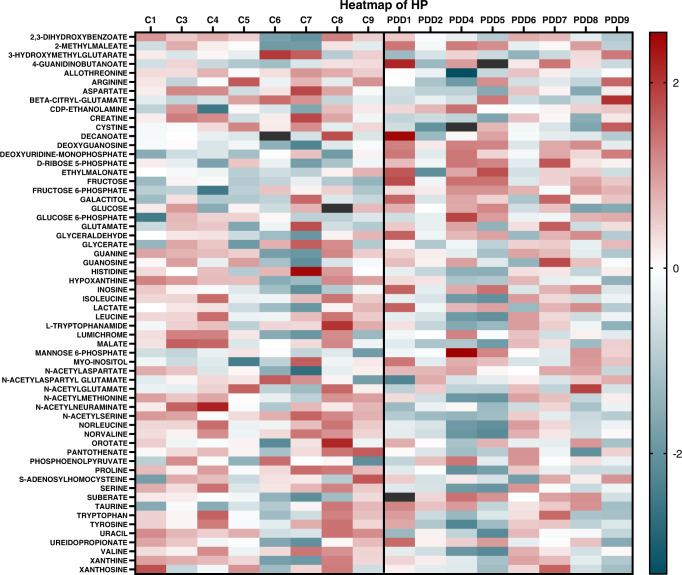
Heat map of metabolites in HP of PDD case and control brains. Heat map created using *z*-score normalised data in GraphPad Prism v9.1.2. All identified metabolites are shown. The darker the colour, the greater the difference in concentration relative to the mean; the red colour denotes increased relative concentrations; the blue colour denotes decreased relative concentrations. Black rectangles denote excluded values. C control, PDD Parkinson’s disease dementia case.

The regions showing the highest number of altered metabolites were the medulla oblongata (MED) and pons (25 each), followed by the HP and middle temporal gyrus (MTG; 24 each; see Fig. 2 and Table 1). The regions with the lowest number of altered metabolites were the motor cortex (MCX; 8), primary visual cortex (PVC; 12) and cingulate gyrus (CG; 12). The cerebellum (CB) and SN showed intermediate levels of metabolite alterations, with 18 each. The number of overall metabolite changes appeared to generally correlate with the stage at which different brain regions are considered to start showing Lewy body deposition according to traditional α-synuclein Braak staging criteria, with regions affected earlier in PDD showing higher numbers of metabolic alterations (see Fig. 2). Despite this trend, the CB showed a similar number of changes to the SN, despite being considered to be relatively unaffected in PDD. The highest number of metabolite decreases was observed in the HP (16), whereas the highest number of increased metabolites was found in the PONS (15).

**Table 1:** Significantly altered metabolites in the PDD brain.

Analyte	CG	CB	HP	MCX	MED	MTG	PONS	SN	PVC
*Amino acids*									
Allothreonine	0.6	**0.4**	**0.5**	0.5	**0.6**	0.6	0.7	0.9	0.7
Aspartate	0.6	0.7	**0.5**	0.6	0.8	0.7	**0.7**	1.0	0.5
Creatine	0.6	0.8	**0.5**	0.7	0.8	0.7	**0.7**	1.1	0.6
Glutamate	1.1	1.1	1.1	1.1	1.2	1.0	1.1	**1.7**	1.1
Histidine	0.8	0.8	**0.5**	0.6	0.7	0.6	**0.7**	1.9	0.6
Isoleucine	0.6	**0.5**	**0.6**	0.6	**0.6**	**0.6**	0.7	**0.5**	0.6
Leucine	0.6	**0.5**	**0.6**	0.6	**0.6**	**0.6**	0.7	**0.5**	0.6
*N*-acetylserine	0.7	**0.5**	**0.4**	0.6	0.7	**0.6**	0.8	0.7	**0.5**
Norleucine	0.6	**0.5**	**0.6**	0.6	**0.6**	**0.6**	0.7	**0.5**	0.6
Norvaline	0.6	**0.5**	**0.6**	0.6	**0.6**	**0.5**	**0.7**	0.7	0.5
Proline	**0.5**	**0.5**	**0.5**	**0.5**	**0.5**	**0.4**	**0.6**	**0.6**	**0.4**
Sarcosine	-	**0.4**	-	-	0.4	**0.3**	0.8	0.9	0.6
Serine	**0.6**	**0.5**	**0.7**	**0.6**	**0.6**	**0.6**	**0.6**	0.7	**0.5**
Tyrosine	0.7	**0.6**	**0.7**	0.7	**0.7**	0.7	0.8	0.7	0.6
Tryptophan	0.8	**0.6**	0.8	0.8	0.7	1.0	0.8	0.6	0.8
Valine	0.6	**0.5**	**0.6**	0.6	**0.6**	**0.5**	**0.7**	0.7	0.5
*Glucose & related metabolite*s									
Fructose	**2.2**	1.6	**2.8**	**2.7**	**2.4**	**2.7**	**2.4**	1.1	**2.8**
Fructose-6-phosphate	**9.9**	0.9	**4.0**	3.2	1.4	**2.3**	1.2	-	1.1
Glucose	1.4	2.0	1.1	1.4	1.3	1.0	**2.1**	0.6	1.0
Glucose-6-phosphate	2.2	0.9	1.5	1.2	-	1.4	**2.3**	-	2.6
Mannose-6-phosphate	**3.6**	1.4	**3.5**	1.6	-	2.5	1.9	3.3	3.6
Ribose-5-phosphate	**2.1**	1.2	**1.9**	1.5	**2.1**	**1.6**	**1.8**	-	**2.3**
*Nucleosides & purine metabolism*									
Deoxyguanosine	**1.7**	**1.5**	**1.8**	**1.8**	**2.1**	**1.7**	**1.5**	**1.5**	**1.6**
Deoxyuridine-monophosphate	**1.6**	1.4	**1.5**	**1.8**	**1.7**	**1.7**	**2.1**	-	2.1
Guanine	0.8	0.8	0.9	0.9	0.9	0.8	0.9	**0.6**	0.9
Guanosine	1.3	**1.9**	1.3	**1.7**	**2.5**	**1.5**	**1.6**	1.2	1.5
Hypoxanthine	0.7	0.8	0.8	0.7	**0.8**	**0.7**	0.8	**0.6**	**0.6**
Inosine	**1.6**	**1.6**	**1.8**	**2.0**	**2.3**	1.7	**1.5**	1.2	**1.8**
Uracil	**0.5**	0.7	**0.6**	0.6	**0.7**	**0.6**	0.8	**0.5**	**0.4**
Ureidopropionate	1.0	0.9	1.1	1.1	**1.2**	1.1	**1.2**	1.3	1.0
Xanthine	0.8	0.8	0.9	0.9	0.9	0.8	0.9	**0.6**	0.8
*TCA & urea cycles*									
Malate	0.6	0.7	**0.6**	0.6	0.7	0.8	0.7	0.9	0.5
*N*-acetylglutamate	0.8	0.9	1.0	0.7	1.0	**0.5**	**0.3**	0.8	**0.6**
*Alternative fuel sources*									
3-hydroxymethyl-glutarate	1.2	1.7	0.8	0.9	1.0	0.5	**0.4**	1.1	0.9
Myo-inositol	1.2	1.3	1.2	1.1	1.2	1.1	1.2	**1.5**	1.3
*Other*									
2,3-dihydroxybenzoate	1.0	0.8	0.9	0.9	0.8	0.8	0.9	**0.6**	0.8
2-methylmalate	1.3	1.0	1.4	1.4	1.3	**1.5**	1.1	0.7	**1.2**
4-guanidinobutanoate	1.4	1.8	1.3	2.3	**2.0**	**1.8**	**2.0**	**2.3**	1.8
Decanoate	-	1.4	1.7	1.3	**1.7**	1.3	1.3	1.1	1.3
Ethylmalonate	**1.9**	1.6	1.6	2.5	**2.0**	**1.9**	**1.7**	1.5	1.5
Galactitol	1.5	**3.1**	1.5	**1.7**	**2.7**	**1.6**	**2.3**	**2.6**	1.4
l-tryptophanamide	0.6	0.6	0.6	0.6	**0.6**	**0.5**	0.7	**0.5**	0.5
Maleate	1.2	-	-	-	-	1.4	-	0.8	**1.2**
*N*-acetylaspartate	1.2	**1.3**	1.2	1.2	**1.2**	1.1	**1.1**	1.3	1.2
*N*-acetylmethionine	0.7	0.6	0.7	0.7	0.7	0.8	1.0	**0.5**	0.7
*N*-acetylneuraminate	0.9	1.1	**0.6**	0.8	1.0	0.8	**1.3**	**2.0**	0.9
Pantothenate	0.6	**0.4**	0.7	0.6	**0.6**	**0.7**	**0.6**	0.6	0.7
*S*-adenosylhomocysteine	0.9	1.3	1.0	0.9	1.1	0.9	1.3	**2.0**	0.8
Suberate	**2.1**	0.9	**1.6**	1.2	1.5	2.1	**2.5**	1.6	1.2

Data shown in the table are fold changes based on non-logged peak area data. Significant case–control fold changes (*p* < 0.05) determined by multiple *t*-tests corrected with 10% FDR are highlighted in bold. Dash indicates samples excluded from analysis due to high-blank values.

Some metabolites were altered in almost every observed region. Fructose was found to be significantly increased in every region except the CB and SN, with increases ranging from 2.2-fold in the CG to 2.8-fold in the HP and PVC. Inosine was altered in every region except the MTG and SN, with increases ranging from 1.5 (pons) to 2.3-fold (MED) in every other region. Serine was significantly decreased in every region except the SN, with fold changes ranging from 0.5-fold in the CB and PVC to 0.7-fold in the HP. Proline was observed to be significantly decreased in every region, with decreases ranging from 0.4-fold in the MTG and PVC to 0.6-fold in the pons and SN. Deoxyguanosine was also altered in every region, with increases ranging from 1.5 (CB and SN) to 2.1-fold (MED).

**Fig. 3:**
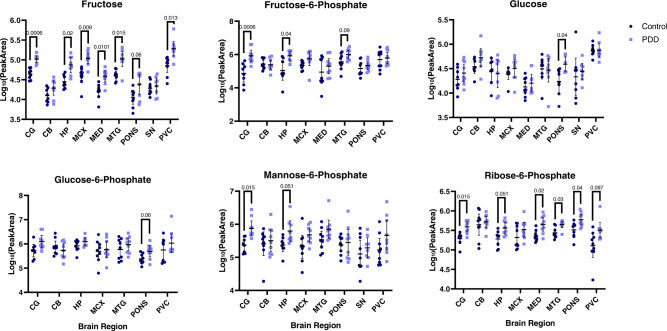
Glucose pathway metabolites in the PDD brain. Data shown are mean log_10_ peak area data ±95% confidence intervals. C control, PDD Parkinson’s disease dementia. *Q* values are shown for significant discoveries determined by multiple *t*-tests corrected by 10% FDR. CB cerebellum, CG cingulate gyrus, HP hippocampus, MCX motor cortex, MED medulla oblongata, MTG middle temporal gyrus, PVC primary visual cortex, SN substantia nigra.

**Fig. 4:**
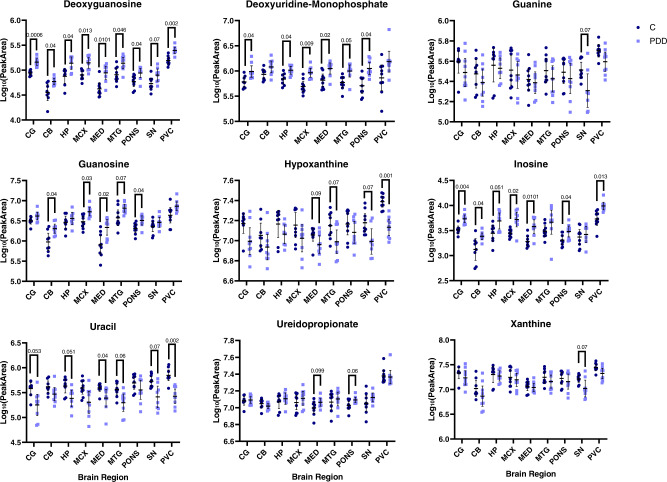
Purine pathway metabolites in the PDD brain. Data shown are mean log_10_ peak area data ±95% confidence intervals. C control, PDD Parkinson’s disease dementia. *Q* values are shown for significant discoveries determined by multiple *t*-tests corrected by 10% FDR. CB cerebellum, CG cingulate gyrus, HP hippocampus, MCX motor cortex, MED medulla oblongata, MTG middle temporal gyrus, PVC primary visual cortex, SN substantia nigra.

The altered metabolites observed in the PDD brain are associated with several metabolic pathways, such as glucose (see Fig. 3) and purine (see Fig. 4) metabolism, as well as including multiple amino acids (see Supplementary Material 1 and Fig. 1 for graphs of all significantly altered metabolites). Multiple metabolites involved in glycolysis were found to be increased in at least one brain region, including glucose, fructose, fructose-6-phosphate and glucose-6-phosphate. Alterations in alternative glucose metabolic pathways were also observed, such as changes in ribose-5-phosphate, which is downstream of glucose-6-phosphate in the pentose phosphate pathway (PPP). As well as glucose pathways, multiple metabolite intermediates of purine metabolism were observed to be altered, including increased guanosine and inosine and decreased hypoxanthine and xanthine.

Principal component analysis (PCA) did not reveal a visual separation of cases and controls with any brain region (see Supplementary Material 1 and Fig. 3).

The nature of the alterations in the identified metabolic pathways varied: significantly altered glucose metabolites were characterised exclusively by upregulation in PDD cases (Fig. 3); purine metabolites were also largely increased, with most exceptions occurring in the SN (Fig. 4), and almost all significantly altered amino acids showed downregulation (Additional File 1 Fig. 2a–c). This reflects a general upregulation of alternative glucose pathways and purine metabolism, along with a generalised downregulation of multiple amino acids.

The type of metabolic pathways affected also varied according to region. The largest number of perturbations in glucose pathways was present in the CG, HP, and PONS (Fig. 3); the highest numbers of altered amino acids were in the HP, MED, and MTG (Additional File 1 Fig. 2a–c); and the most extensive dysregulation of the purine metabolism pathway was found in the MED, PONS and SN (Fig. 4). The CG showed mostly alterations in the glucose and purine pathways, the CB primarily showed amino acid changes, the HP was characterised by the highest observed number of amino acid alterations, the MCX’s few changes were primarily found in the purine pathway, the MED showed extensive perturbations in amino acid and purine metabolism—along with several changes that did not group into a particular metabolic pathway—the MTG primarily showed amino acid alterations, changes in the pons were distributed quite evenly across all pathways, the SN showed moderate changes in both amino acid and purine metabolites, and changes in the PVC were shared fairly evenly among the different identified pathways. The few perturbations found in the TCA and urea cycles were confined to the HP, MTG, pons, and PVC. These varying distributions of metabolic changes may reflect the different vulnerabilities of different PDD brain regions to distinct metabolic insults.

**Fig. 5:**
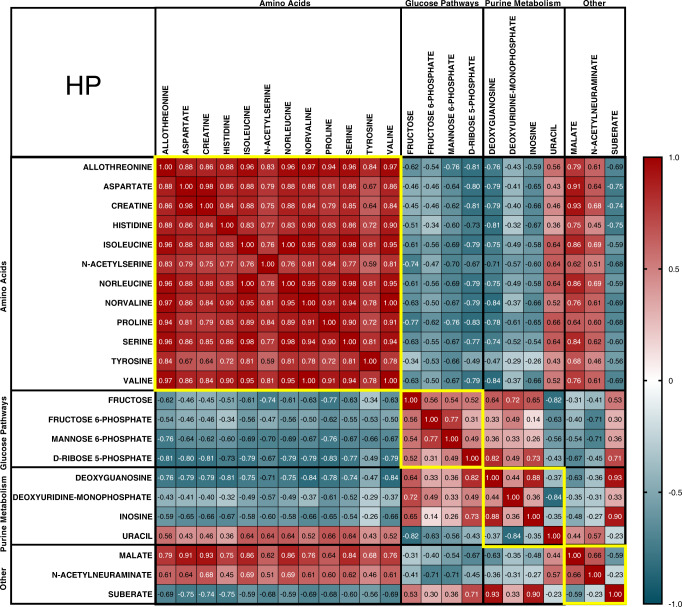
HP metabolite correlation matrix. Matrix shows non-parametric Pearson correlation coefficient *r* values. Significant correlations are defined as *r* > 0.5, *p* < 0.05; *r* = 0.5–0.6 are weak correlations, *r* = 0.6–0.7 are moderate correlations, *r* = 0.7–0.9 are strong correlations, and *r* > 0.9 are very strong correlations. All correlations of *r* < 0.5 had a *p* value >0.05. The red colour denotes positive correlations, while blue denotes negative correlations; the darker the colour, the stronger the correlation.

A non-parametric Pearson correlation analysis was carried out to investigate the relationships between significantly altered metabolites in each brain region; a representative correlation matrix of the HP is shown in Fig. 5, with matrices for other regions included in Additional File 1 and Fig. 4. Although correlations varied between different regions, there were some general patterns: amino acids were generally highly, positively correlated—often with Pearson’s coefficients of 0.8 or higher—with several showing coefficients >0.9. Conversely, amino acids generally showed negative correlations with metabolites in other groups, with a few exceptions—most of which were organic acids, such as pantothenate and malate.

Metabolites included in the glucose and purine metabolism groups generally showed moderate (*r* = 0.6–0.7), strong (*r* = 0.7–0.9) or very strong (*r* > 0.9) positive correlations with each other; exceptions included hypoxanthine and uracil, which generally showed either no correlation (*r* < 0.5; *p* > 0.05) or weak to moderate negative correlations with other metabolites in these two groups. Uracil and hypoxanthine did show positive correlations of varying strengths with most amino acid metabolites, but otherwise showed no correlation or weak to moderate negative correlations with other metabolites.

Metabolites in groups outside the amino acid, glucose and purine metabolism groups (designated ‘Other’ on correlation matrices) tended to show more varying relationships with one another; however, there were still some general patterns—those that showed positive correlations with metabolites in the amino acid group often showed negative correlations with glucose and purine metabolism metabolites, or vice versa. Generally, the correlation coefficients for metabolites in the ‘Other’ group tended to be either non-significant or of low to moderate strength, with few exceptions.

## Discussion

To the authors’ knowledge, this study presents the most comprehensive systematic, semi-targeted metabolomics analysis across multiple regions of the PDD brain, identifying 49 metabolic alterations across nine investigated brain regions, including multiple amino acids as well as metabolites involved in glucose and purine metabolism. The number of alterations varied depending on the brain region investigated, with regions affected earlier in the disease (as described by α-synuclein Braak staging^16^) showing a higher number of changes. This suggests that metabolic disruptions reflect the temporal pattern of neuropathology in PDD, rather than the degree of neuronal loss in different regions. One exception to this rule appears to be the CB, which showed a moderate number of metabolite alterations despite traditionally being considered to be relatively unaffected by neurodegeneration in PD/PDD. This may indicate a greater involvement of the CB in PDD than previously thought. Indeed, Lewy body deposition and dopaminergic neurodegeneration has been observed in the CB in PD, despite its lack of involvement in the description of typical α-synuclein Braak stage progression^17–20^.

### Glucose metabolism

**Fig. 6:**
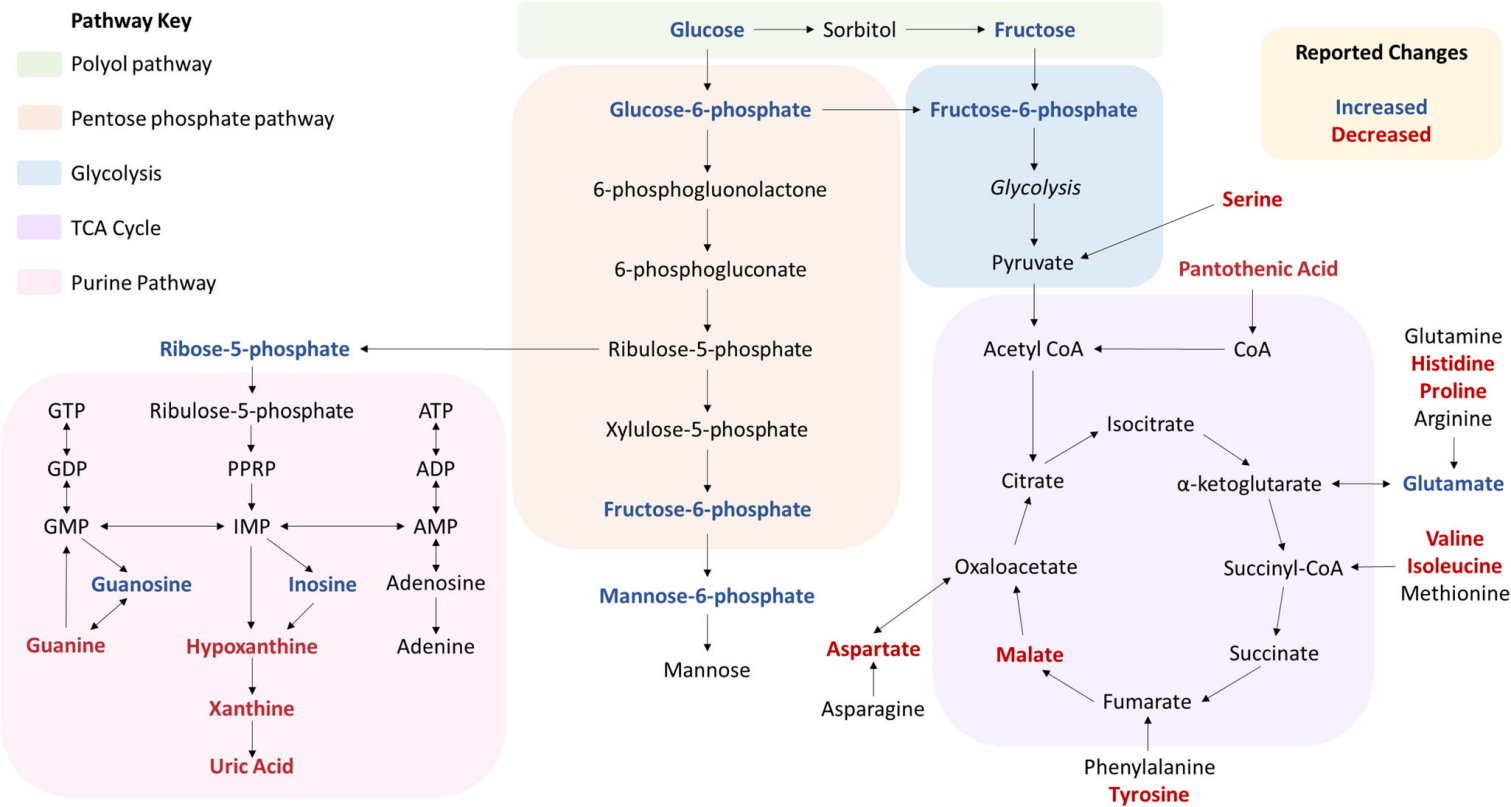
Pathway changes in the PDD brain. The figure indicates changes found in at least one investigated PDD brain region. ADP adenosine diphosphate, AMP adenosine monophosphate, ATP adenosine triphosphate, GMP guanosine monophosphate, GTP guanosine triphosphate, IMP inosinic acid, PPRP phosphoribosyl pyrophosphate.

Evidence for impaired glucose metabolism has been reported extensively in the PD and PDD brain^21–23^, with some previous reports of increased glucose^4^ and decreased glucose-6-phosphate^5^ levels in the PD cortex. The cortex as a whole was not investigated in this study, but increases in glucose were identified in the pons, alongside increases in glucose-6-phosphate. Strikingly, fructose showed increases of up to 2.8-fold in all investigated regions except the CB and SN, and fructose-6-phosphate also showed large increases of up to 9.9-fold in the CG, HP and MTG. Fructose elevations, in combination with widespread elevations in ribose-5-phosphate, and more limited increases in glucose-6-phosphate and fructose-6-phosphate, may indicate increased activity of the polyol pathway and pentose phosphate pathway and decreased glycolytic activity in PDD (see Fig. 6). Indeed, increased pentose phosphate pathway activity has been previously observed in the PD cortex and putamen, in the late stages of the disease^5^ and drug-induced increases (e.g. with terazosin) of glycolysis have been shown to slow the progression of parkinsonism and dopaminergic cell loss in PD animal models; individuals taking such drugs for non-PD-related conditions show a reduced incidence of PD^24^. The relative changes in these different glucose metabolism pathways may contribute to mitochondrial dysfunction, glucose hypometabolism and energy production deficits in the PDD brain.

### Purine metabolism

Purines are essential components of the DNA and RNA nucleobase backbone and also function as components of other metabolic molecules that are involved in a great number of metabolic pathways, such as adenosine triphosphate (ATP), cyclic adenosine monophosphate (cAMP), reduced nicotinamide adenine dinucleotide (NADH) and coenzyme A (CoA); disruptions in the purine metabolic pathway (see Fig. 6) may lead to insufficient pools of purine components for these pathways. Here, we report several alterations in purine metabolism, including widespread increases in inosine—in the CB, CG, HP, MCX, MED, PONS and PVC—increased deoxyguanosine in every investigated region, region-specific increases in guanosine, deoxyuridine-monophosphate and ureidopropionate, and localised decreases in guanine, hypoxanthine, uracil and xanthine. Increased adenosine and inosine, as well as decreased uric acid, have been previously reported in the PD cortex and striatum of males, with female-specific increases in xanthine^7^, although such sex-specific differences were not apparent in the current PDD cohort. Together, these observations suggest substantial disruption of the purine metabolism pathway in the PDD brain (see Fig. 6).

### Amino acids

Multiple amino acids were observed to be altered in the PDD brain, with widespread decreases in serine and proline affecting every investigated region (except for the SN in the case of serine), as well as region-specific decreases in isoleucine, valine and histidine, among others. MTG-specific increases in leucine and isoleucine were also present. Amino acids provide substrates to multiple metabolic pathways, including the TCA cycle (see Fig. 6); diminished amino acid levels may prevent the proper functioning of these pathways. Indeed, some alterations in the TCA cycle have previously been observed in the PD brain, including decreases in the activity of the rate-limiting enzyme α-ketoglutarate dehydrogenase in the PD CB^8^; however, most studies report only peripheral levels^25^. Amino acids may influence neurotransmitter synthesis and functioning, as many are precursor molecules of different neurotransmitters, such as tyrosine and glutamate^26^. Here, tyrosine was observed to be decreased in the HP, MED and CB of PD brains, which may exacerbate the disease-defining loss of dopamine in PD/PDD. Glutamate, which was increased in the PDD SN, is the precursor of the inhibitory neurotransmitter γ-aminobutyric acid (GABA), which has been reported to be increased in the basal ganglia^27,28^ and PUT^29^ in PD. However, other reports have observed decreased GABA levels in the PD basal ganglia^30^, as well as in the occipital lobe of PD patients experiencing visual hallucinations^31^. As such, findings concerning GABA levels in PD require further validation.

### Other

Alterations in several other metabolites were also identified here in the PDD brain. The most widespread alterations were in galactitol, which was observed to be elevated in all regions except the CG, HP and PVC, with increases of up to 3.1-fold. Pantothenic acid was observed to be decreased in the CB, MED, MTG and pons of PDD cases here, similar to the results found in a previous targeted analysis of pantothenic acid in PDD brains carried out by our group^32^. Pantothenic acid is essential to the formation of CoA, which is involved in almost all metabolic pathways, including the TCA cycle and glycolysis (see Fig. 4). Decreased pantothenic acid levels may lead to insufficient levels of CoA being produced, as has been previously observed in the PD brain^8^, with potential downstream effects on the metabolic pathways in which it is involved.

### Strengths and limitations of the study

The primary limitation of this study is the relatively small sample size employed, which may increase the number of type II statistical errors when investigating large numbers of metabolites. This may have also contributed to the lack of separation observed in the PCA plots, despite a number of statistically significant results being observed in individual metabolites. The current sample size was selected based on previous experiments in which these numbers were sufficient to obtain significant case–control results in AD and HD brains^33,34^—many of which have been replicable in further targeted studies and investigations performed by other groups^35–39^. However, the results of future studies would be greatly strengthened with the use of more robust sample sizes—although this can be difficult in studies of the brain due to the limited availability of suitable tissues. Alternatively, targeted, quantitative analyses of the analytes of interest identified here would be at less risk of such type II errors; such quantitation is not feasible when looking at high numbers of analytes simultaneously. As such, the primary purpose of the results obtained in this study is to act as a discovery dataset, highlighting analytes of interest for future, more targeted studies. Additionally, the methodology employed in this study involved the use of whole tissue, which gives no information on the intra/extracellular or subcellular localisation of the analytes studied. As such, we have no data on alterations in the localisation of significantly altered analytes, and analytes which showed no significant changes in whole tissue may have shown alterations in specific subcellular compartments or relative changes between the intra/extracellular environment should the methodology have allowed this.

Concerning the tissues used, one strength of this study was the restriction of PMD to 26 h or less. Although it is difficult at times to obtain brain tissues with low PMD due to legal and practical constraints in the harvesting of such samples, a previous pilot study conducted by ourselves found that the concentrations of many metabolites altered in the brains of healthy rats over the course of 72 h—with many already showing some changes by as little as 24 h^40^. As such, we took precautions to obtain as low a PMD as possible in the tissues available, as close to 24 h as possible, while retaining strict age- and gender-matching. Additionally, when selecting the cohort, a deliberate distinction was made between PD with and without dementia, whereas most PD cohorts are either mixed or do not distinguish between PD cases with and without cognitive decline; this could allow for comparisons of PD and PDD in potential future studies. The greatest strength of this study is the analysis of ten brain regions obtained from the same cohort, covering areas of the brain with varying levels of neurodegeneration in HD. As such, this study has some of the most extensive coverage of PDD brain metabolomics in the current literature.

Overall, this report represents a comprehensive systematic, multi-regional, semi-targeted metabolic investigation of the PDD brain—highlighting several metabolic pathways for future targeted studies and potential therapeutic targets, including glucose and purine metabolism, among others. The number of these alterations reflected the α-synuclein Braak stage in which these brain regions are first affected by Lewy body deposition in traditional PD Braak staging, indicating that metabolic changes may reflect the temporal spread of PD neuropathology rather than the degree of neuronal loss in PDD.

## Methods

### Obtaining tissue for LC–MS metabolomics

Tissues were obtained from nine brain regions, including the middle temporal gyrus (MTG); motor cortex (MCX); primary visual cortex (PVC); hippocampus (HP); anterior cingulate gyrus (CG); cerebellum, at the level of the dentate nucleus (CB); SN; pons and medulla oblongata (MED). These regions were selected in order to cover areas of the brain that are usually considered to be severely and moderately affected, as well as relatively spared in PDD; having these regions allows for the investigation of whether metabolic alterations in the PDD brain are localised or widespread, and to identify whether changes in particular metabolic pathways are localised to areas of the brain associated with different PDD symptoms (e.g. motor dysfunction or cognitive decline).

Tissues were obtained from nine cases with neuropathologically-confirmed diagnoses of PDD and eight controls from the Miami Brain Endowment Bank, Miami, FL, USA (part of the National Institute of Health NeuroBioBank network). All patient metadata available from the NeuroBioBank, including the cause of death, comorbidities and neuropathological findings, were obtained and are presented in Supplementary Material 1 and Table 1. Information regarding the collection, processing, and neuropathological examination of tissues at the NIH NeuroBioBank can be found at https://neurobiobank.nih.gov/about-best-practices/.

### Ethics and consent

All cases included in the NIH NeuroBioBank database were collected for research purposes with written informed consent from all donors and/or next of kin, with approval from the designated Institutional Review Board of each individual contributory brain bank. This study and the use of the tissues was approved by Manchester REC (09/H0906/52 + 5).

### Diagnosis and severity of PDD cases

Board-certified neuropathologists at the Miami Brain Endowment Bank diagnosed all donor tissue. All PDD cases were diagnosed to be the α-synucleinopathy neocortical type, consistent with the clinical phenotype of PDD. Controls did not show any neuropathological or clinical features of neurodegenerative disease or vascular pathology. Brains were assessed according to standardised diagnostic criteria, e.g. α-synuclein Braak staging^16^ and/or McKeith’s^41^ staging criteria for Lewy body-related neuropathology (see Supplementary Material 1 and Table 1 for individual data) and the 2012 National Institute on Aging-Alzheimer’s Association Guidelines for AD^42^ to assess for AD-related amyloid and tau pathology. All available clinical data, including medical records, autopsy reports, family interviews, etc., were reviewed by clinicians at the NIH NeuroBioBank in order to ascertain clinical diagnoses and comorbidities. Unfortunately, data regarding the severity of the cognitive decline in cases was not available from the brain bank.

### Tissue dissection

Brain samples were cut into sections of 50 mg (±5 mg) for HPLC–MS analysis using a metal-free ceramic scalpel. Samples were stored in ‘Safe-Lok’ microfuge Eppendorf tubes (Eppendorf AG; Hamburg, Germany) and stored at −80 °C prior to extraction.

### HPHPLC–MS metabolomics

Untargeted metabolic analysis was carried out on the brain samples using HPLC–MS. Samples were extracted in 800 µl 50:50 (v/v) methanol:chloroform before being lysed in a TissueLyser batch bead homogeniser (Qiagen, Manchester, UK) with a 3 mm carbamide beads at 25 Hz for 10 min. Blanks were also prepared containing only 800 µl 50:50 (v/v) methanol:chloroform. Following lysing, 200 µl of HPLC–MS grade water was added to samples before centrifugation at 2400×*g* for 15 min for separation of polar and non-polar phases. About 200 µl of the polar methanol phase was transferred to a new tube before being dried overnight in a Speedvac centrifugal concentrator (Savant Speedvac, Thermo Scientific, UK) at ~25 °C for 16–18 h. Samples were resuspended in 100 µl acetonitrile and water in a ratio of 5:1. The sample was then centrifuged at 20,000×*g* for 3 min and the top 80 µl was transferred to a glass autosampler vial with a 300 µl insert and capped.

HPLC–MS analysis was performed using a Thermo Fisher Ultimate 3000 HPLC system consisting of an HPG-3400RS high-pressure gradient pump, TCC 3000 SD column compartment and WPS 3000 Autosampler (Thermo Fisher, Waltham MA, USA), coupled to a SCIEX 6600 TripleTOF Q-TOF mass spectrometer with TurboV ion source (Sciex, Framingham MA, USA). The system was controlled by SCIEX Analyst 1.7.1 (Sciex), DCMS Link (Thermo Fisher) and Chromeleon Xpress software (Thermo Fisher).

A sample volume of 5 μL was injected by pulled loop onto a 5 μL sample loop with 150 μl post-injection needle wash with 9:1 acetonitrile and water. The injection cycle time was 1 min per sample. Separations were performed using an Agilent Poroshell 120 HILIC-Z PEEK-lined column with dimensions of 150 mm length, 2.1 mm diameter and 2.7-μm particle size equipped with a guard column of the same phase (Agilent, Santa Clara, CA, USA). Mobile phase A was water with 10 mM ammonium acetate adjusted to pH 9 with ammonium hydroxide and 20 µM medronic acid, mobile phase B was 85:15 acetonitrile and water with 10 mM ammonium acetate adjusted to pH 9 with ammonium hydroxide and 20 µM medronic acid. Separation was performed by gradient chromatography at a flow rate of 0.25 ml/min, starting at 96% B for 2 min, ramping to 65% B over 20 min, holding at 65% B for 2 min, then back to 96% B. Re-equilibration time was 5 min. Total run time, including 1 min injection cycle was 30 min.

The mass spectrometer was run in the negative mode under the following source conditions: curtain gas pressure, 50 psi; ionspray voltage, −4500 V; temperature, 400 °C; ESI nebuliser gas pressure, 50 psi; heater gas pressure, −70 psi; declustering potential, −80 V.

Data were acquired in a data-independent manner using SWATH in the range of 50–1000 m/z, split across 78 variable-size windows (79 experiments including TOF survey scan), each with an accumulation time of 20 ms. The total cycle time was 1.66 s. The collision energy of each SWATH window was determined using the formula CE (V) = 0.084 x m/z + 12 up to a maximum of 55 V. Samples were given a non-classifying label at the beginning of the experiment, run in a randomised order, and run with the investigator blinded to disease status.

### Data analysis

The acquired data were processed in MultiQuant 3.0.2 (Sciex). Peaks from the MS1 and MS2 data were picked and matched against a metabolite library of 235 standards based on retention time and mass error of ±0.025 Da. Data exported from MultiQuant 3.0.2 was further sorted, filtered, and scored using a custom VBA macro in Excel based on presence, peak area and the coelution of precursor and fragment ions.

The statistical significance of individual metabolite peak areas was calculated using multiple two-tailed *t*-tests, corrected for the potential effects of multiple comparisons by applying a false-discovery rate (FDR) of 10% (*q* value <0.1) using the two-stage Benjamini, Krieger, and Yekutieli correction in GraphPad v9.1.2 (Prism; La Jolla, CA). Where blank values were >10% of the lowest sample value, the analyte was removed from the analysis. Analytes with a coefficient of variance of >30% in quality controls were also excluded from the analysis. Multiple *t*-tests were applied to each brain region individually (resulting in 55–63 *t*-tests per region, after the exclusion of high-blank metabolites). For graphs, peak areas were transformed by log_10_ and *q* values are shown. A list of all identified signals is included in Supplementary Material 2, along with fold changes, *p* values and *q* values. Fold changes between cases and controls were determined using non-logged peak area data. Heat maps of all metabolite fold changes in each investigated region were created in GraphPad v9.1.2. using *z*-score-normalised data.

PCA analyses were carried out in MetaboAnalyst, with normalisation by a median, log transformation and auto-scaling (mean-centred and divided by the standard deviation of each variable) for each individual region. Correlations between significantly altered metabolites were determined using non-parametric Spearman correlation coefficients in GraphPad v.9.1.2; correlations were classed as non-significant (*r* < 0.5; *p* > 0.05), weak (*r* = 0.5–06), moderate (*r* = 0.6–0.7), strong (*r* = 0.7–0.9) or very strong (*r* > 0.9).

### Reporting summary

Further information on research design is available in the Nature Research Reporting Summary linked to this article.

## Supplementary information

Supplementary Information 1

Reporting Summary

Supplementary Information 2 - Raw Data

## Data Availability

The datasets supporting the conclusions of this article are included within the article (and its Supplementary Material(s).
