# Supplementary material for: Multi-regional alterations in glucose and purine metabolic pathways in the Parkinson’s disease dementia brain
Source: NPJ Parkinsons Dis. 2023 Apr 20;9:66. doi: 10.1038/s41531-023-00488-y (PMC10119289; doi:10.1038/s41531-023-00488-y)
Supplement: Supplementary file 1 — Supplementary Information 1 [file 41531_2023_488_MOESM1_ESM.pdf]

# Supplementary Material A

## Contents


Additional File Table 1: Characteristics of individuals in the PDD Metabolomics cohort ..... 3

Additional File Table 2: Summary of PDD Metabolomics cohort ..... 6

Additional File Figure 1A – Significant Altered Metabolites in PDD Brain: Amino Acids 1 ..... 7

Additional File Figure 2B – Significant Altered Metabolites in PDD Brain: Amino Acids 2 ..... 8

Additional File Figure 1C – Significant Altered Metabolites in PDD Brain: Glucose Metabolism Pathways ..... 9

Additional File Figure 1D – Significant Altered Metabolites in PDD Brain: Purine Metabolism Pathways ..... 10

Additional File Figure 1F – Significant Altered Metabolites in PDD Brain: Other Metabolites ..... 11

Additional File Figure 2A – Heat Map: CG ..... 13

Additional File Figure 2B – Heat Map: CB ..... 14

Additional File Figure 2C – Heat Map: MCX ..... 15

Additional File Figure 2D – Heat Map: MED ..... 16

Additional File Figure 2E – Heat Map: MTG ..... 17

Additional File Figure 2F – Heat Map: Pons ..... 18

Additional File Figure 2G – Heat Map: SN ..... 19

Additional File Figure 2H – Heat Map: PVC ..... 20

Additional File Figure 3 – PCA Plots ........ 21
Additional File Figure 4A – Correlation Matrix: CG ........ 22
Additional File Figure 4B – Correlation Matrix: CB ........ 23
Additional File Figure 4C – Correlation Matrix: MCX ........ 24
Additional File Figure 4D – Correlation Matrix: MED ........ 25
Additional File Figure 4E – Correlation Matrix: MTG ........ 26
Additional File Figure 4F – Correlation Matrix: Pons ........ 27
Additional File Figure 4G – Correlation Matrix: SN ........ 28
Additional File Figure 4H – Correlation Matrix: PVC ........ 29

Additional File Table 1: Characteristics of individuals in the PDD Metabolomics cohort

| Code | Sex | Age at death | Clinical diagnosis | Neuropathological Findings | Post-mortem Delay (hours) | Whole-brain weight (g) | Cause of Death |
|---|---|---|---|---|---|---|---|
| C1 (no SN tissue) | Male | 61 | No dementia present | Normal adult brain | 12.5 | 1182 | Respiratory failure; heart failure; coronary artery disease |
| C2 | Male | 71 | No dementia present | Normal adult brain | 23.9 | 1371 | Acute myocardial infarction; severe coronary artery disease; pulmonary oedema; diabetes mellitus; cardiopulmonary arrest |
| C3 | Male | 74 | No dementia present | Unaffected control with incidental Lewy bodies | 25.5 | 1300 | Aortic dissection |
| C4 (no SN tissue) | Male | 70 | No dementia present | Low level Alzheimer's disease neuropathological change (B0, C1) | 12.7 | 1350 | Atherosclerotic and hypertensive heart disease |
| C5 | Female | 68 | No dementia present | Normal adult brain | 19.1 | 1270 | Acute myocardial infarction; coronary artery disease |
| C6 | Female | 65 | No dementia present | Normal adult brain with minimal small vessel ischaemic changes | 19.4 | 1372 | Hypertensive arteriosclerotic cardiovascular disease; morbid obesity; respiratory arrest; suspected embolus |
| C7 | Female | 77 | No dementia present | Normal adult brain | 21.4 | 1135 | Pending death certificate |

| | | | | | | | |
|---|---|---|---|---|---|---|---|
| C8 | Female | 79 | No dementia present | Normal adult brain | 17.8 | 1300 | Acute myocardial infarction; coronary artery disease; atrial fibrillation; COPD |
| C9 | Female | 67 | No dementia present | Alzheimer's disease neuropathological changes (A1, B1, C0), low level normal aging, cerebrovascular disease, focal minimal atherosclerosis, congestion, oedema, perivascular haemorrhage | 25.9 | 1382 | Pending death certificate |
| C10 (SN ONLY) | Male | 68 | No dementia present | Normal adult brain | 10.8 | 1550 | Pending death certificate |
| C11 (SN ONLY) | Male | 62 | No dementia present | Normal adult brain; capillary haemangioma in caudate nucleus | 21.3 | Not available | Diabetes; triple vessel disease of the heart; hyperlipidaemia |
| PDD1 | Male | 61 | Parkinson's disease dementia | Limbic (transitional) LBD | 13.8 | 1382 | Cardiopulmonary arrest; probable acute myocardial infarction; Parkinson's disease |
| PDD2 | Male | 79 | Parkinson's disease dementia | Limbic (transitional) LBD | 16.2 | 1188 | End stage Parkinson's disease |
| PDD3 | Male | 71 | Parkinson's disease | Brainstem predominant LBD; a-synuclein Braak | 16.2 | 1250 | Pending death certificate |

| | | | | | | | |
|---|---|---|---|---|---|---|---|
| | | | dementia | stage III-IV | | | |
| PDD4 | Male | 78 | Parkinson's disease dementia | Diffuse neocortical LBD; a-synuclein Braak stage VI; low level of AD neuropathic change (B1, C1) | 20.4 | 1520 | Respiratory failure; aspiration pneumonia; dysphagia; Parkinson's disease |
| PDD5 | Male | 70 | Parkinson's disease dementia | Diffuse neocortical LBD | 4.3 | 1218 | Aspiration pneumonia; Parkinson's disease |
| PDD6 | Female | 69 | Parkinson's disease dementia | Brain with morphological findings most compatible with PD with dementia; a-synuclein Braak stage IV-V | 17.5 | 1218 | Pending death certificate |
| PDD7 | Female | 81 | Parkinson's disease dementia | Diffuse neocortical LBD; low level Alzheimer's neuropathological change in limbic system (B1, C1) | 7.0 | 1187 | End stage Parkinson's disease |
| PDD8 | Female | 79 | Parkinson's disease dementia | Limbic (transitional) LBD; mild small vessel ischaemic disease | 21.9 | 1415 | Respiratory failure; Parkinson's disease |
| PDD9 | Female | 67 | Parkinson's disease dementia | Diffuse neocortical LBD; low level Alzheimer's neuropathological change (A3, B1, C1, Braak II) | 14.5 | 1200 | Lewy body disease; Parkinson's disease |

## Additional File Table 2: Summary of PDD Metabolomics cohort

| | Gender (% male) | Age at death (years) | PMD (hours) | Brain Weight (g) |
|---|---|---|---|---|
| **Controls (n = 9)** | 38 | 70 (65–79) | 19.8 (12.7–25.9) | 1296 (1135–1382) |
| **SN Controls (n = 9)** | 44 | 70 (62–79) | 20.6 (10.8–25.9)* | 1335 (1135–1550)† |
| **Cases (n = 9)** | 66 | 73 (61–81) | 14.6 (4.3–21.9) | 1286 (1187–1520) |

*Table shows mean (range) age, PMD, brain weight and Braak stage. †Brain weight unavailable for C10. * SN controls had higher PMD than SN cases; p = 0.03.*

Brain tissue for all nine investigated regions was obtained from the same case and control donors, with the exception of the SN, for which two control samples had to be substituted due to a lack of available tissue in the standard cohort donors (controls C10 and C11 substituted for C1 and C4; see Table 1 for individual data). PDD cases and controls for all regions except the SN showed no significant difference in age, gender distribution, or brain weight; PMD was lower in cases (mean 14.6 hours) compared to controls (mean 20.6 hours; p = 0.03) in the SN, but matched in all other regions. Due to an unavailability of tissue or high blank values, the C1 CG, C5 CB, C2 HP, and PDD3 HP could not be analysed; these regions remained age, gender, brain weight, and PMD-matched. The PMD was kept as low as possible, at a maximum of 25.9 hours in any region and at an average of 17.1 hours.

Additional File Figure 1A – Significant Altered Metabolites in PDD Brain: Amino Acids 1

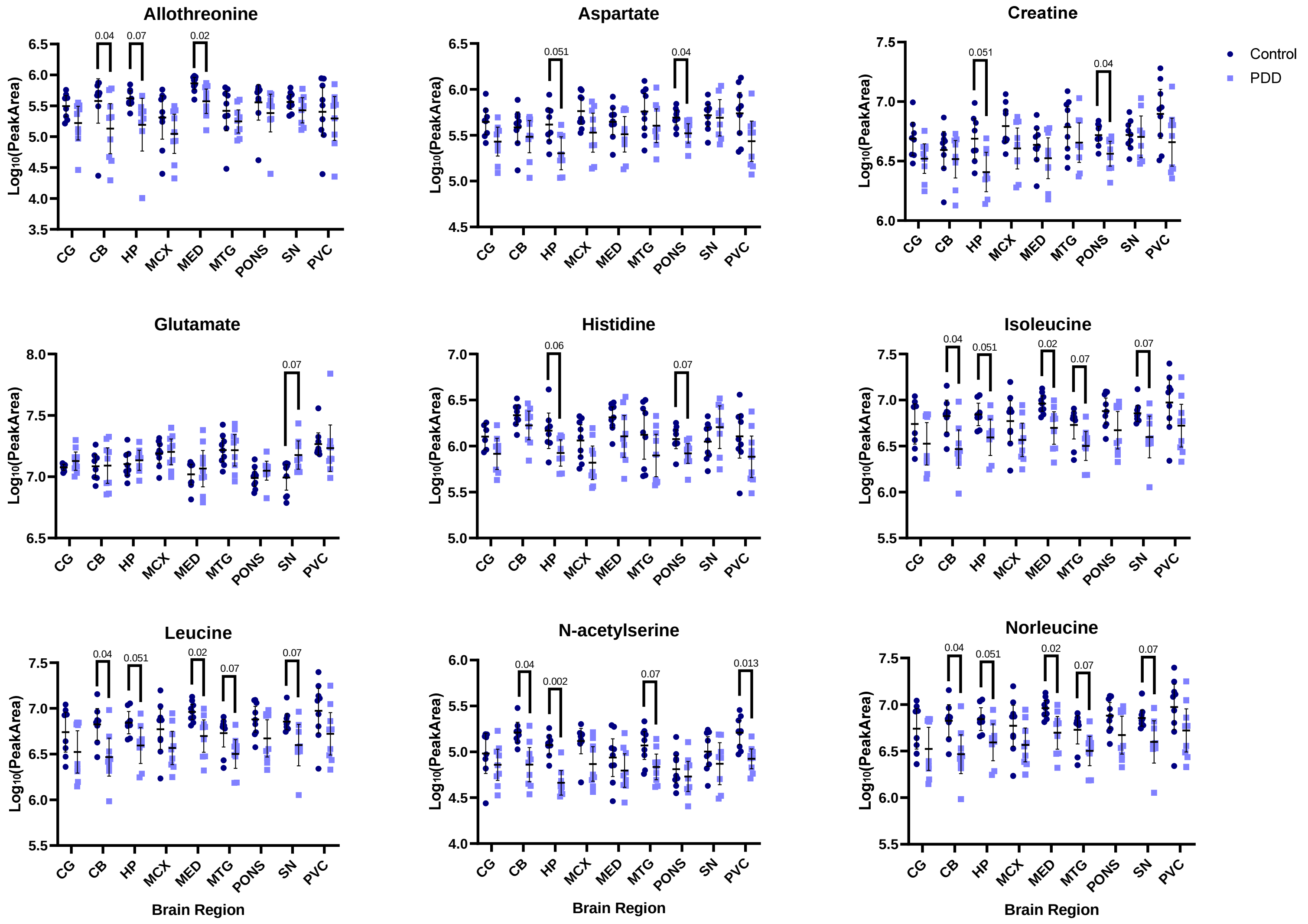

# Additional File Figure 2B – Significant Altered Metabolites in PDD Brain: Amino Acids 2

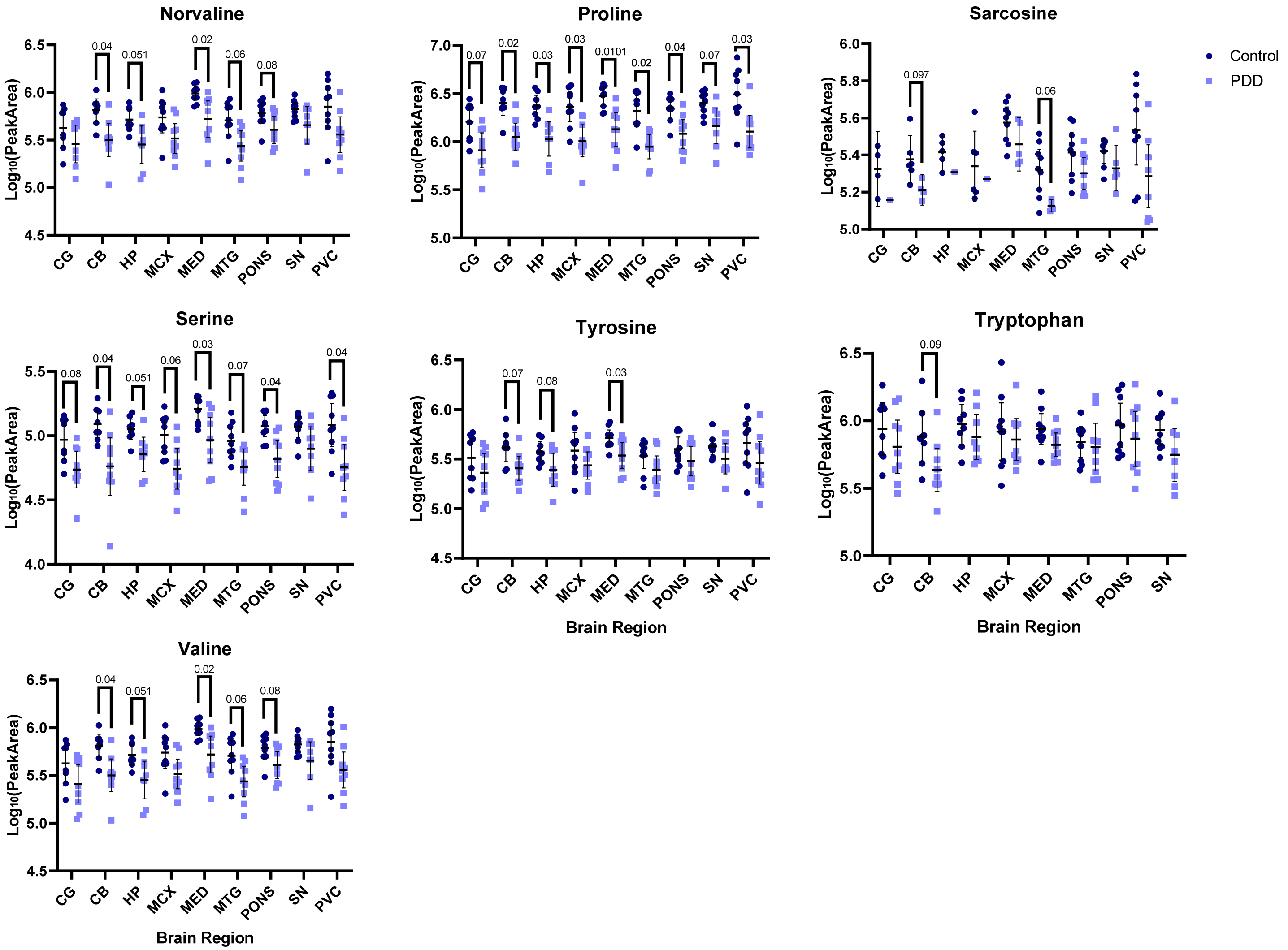

## Additional File Figure 3C – Significant Altered Metabolites in PDD Brain: Glucose Metabolism Pathways

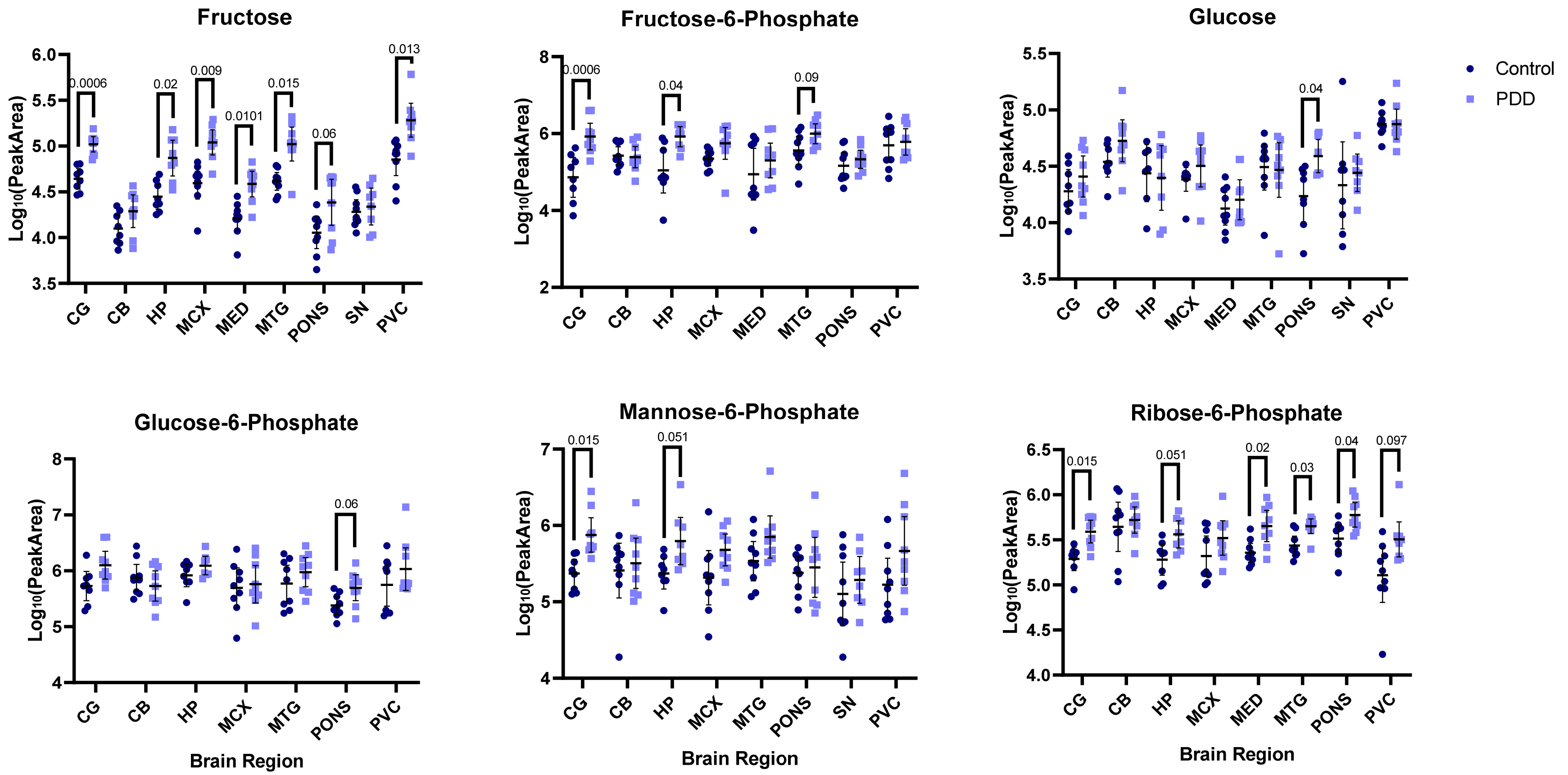

# Additional File Figure 4D – Significant Altered Metabolites in PDD Brain: Purine Metabolism Pathways

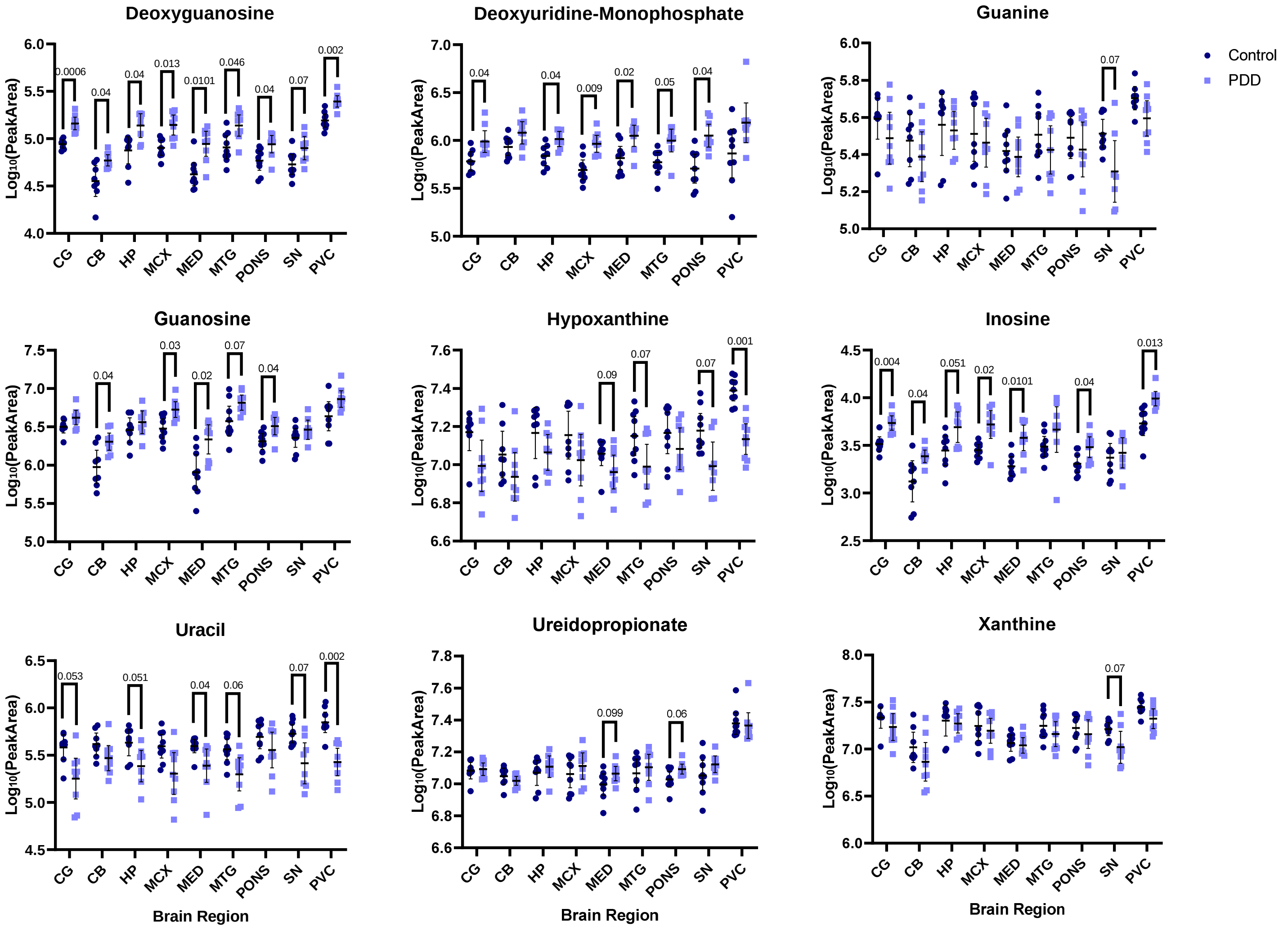

# Additional File Figure 5F – Significant Altered Metabolites in PDD Brain: Other Metabolites 1

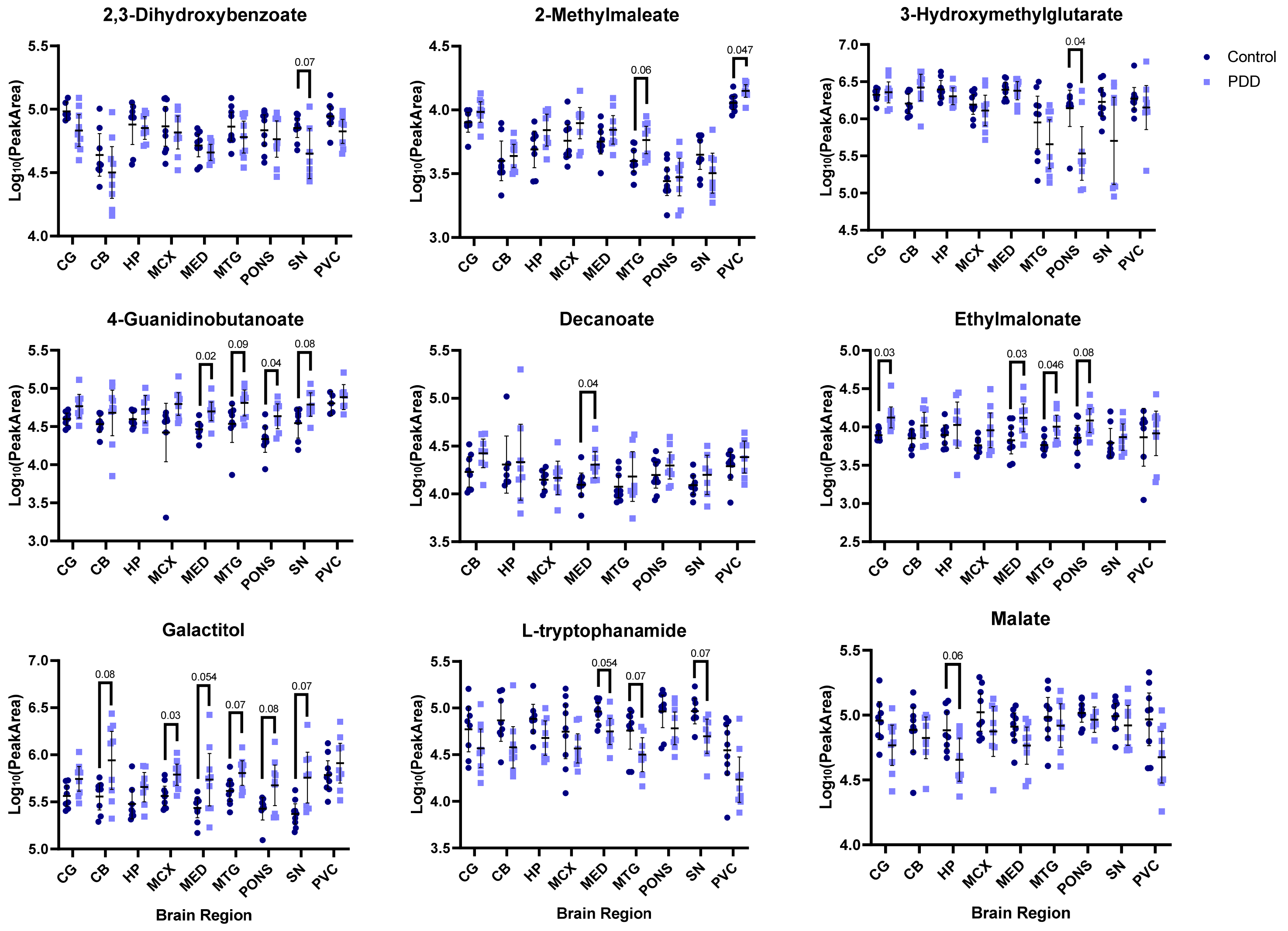

## Additional File Figure 6G – Significant Altered Metabolites in PDD Brain: Other Metabolites 2

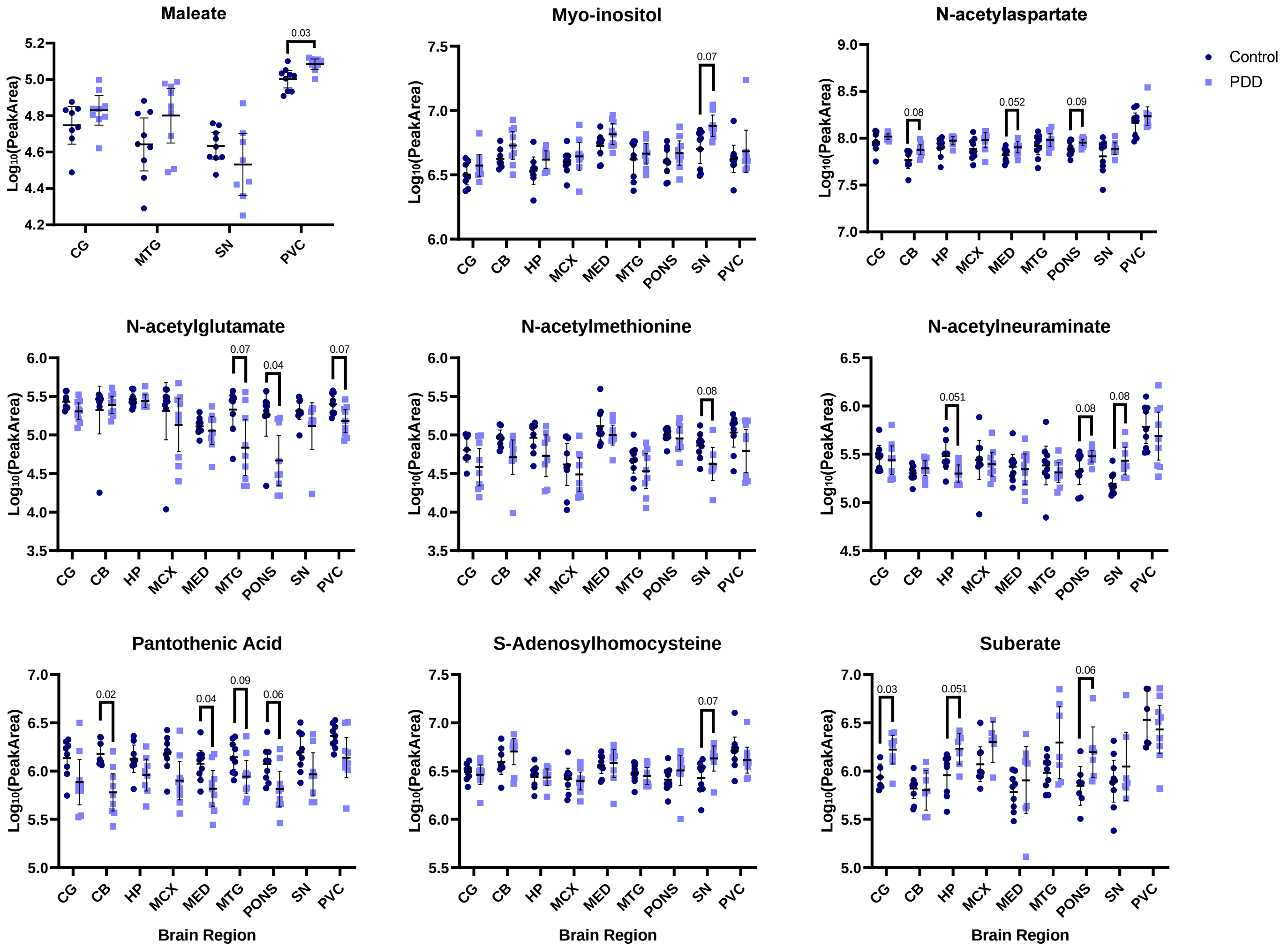


Figure 5A-G. Graphs show $log_{10}$ peak area data ± 95% confidence intervals. Q-values are shown for discoveries following multiple t-tests with 10% FDR correction.

# Additional File Figure 2A – Heat Map: CG

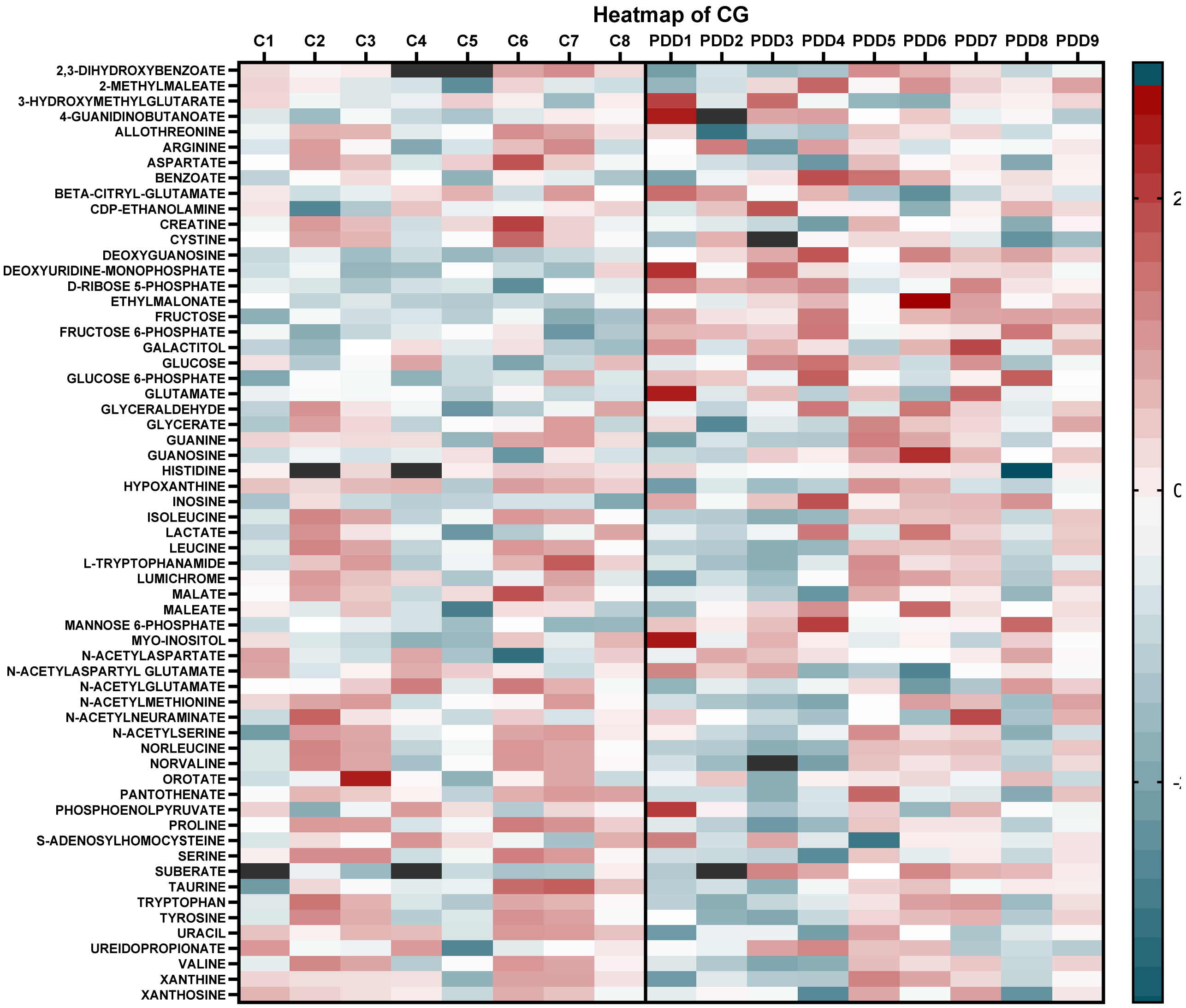


Heat map created using z-score normalised data in GraphPad Prism v9.1.2. All identified metabolites are shown. The darker the colour, the greater the difference in concentration relative to the mean; the red colour denotes increased relative concentrations; the blue colour denotes decreased relative concentrations. Black rectangles denote excluded values. C = control; PDD = Parkinson's disease dementia case.

# Additional File Figure 2B – Heat Map: CB

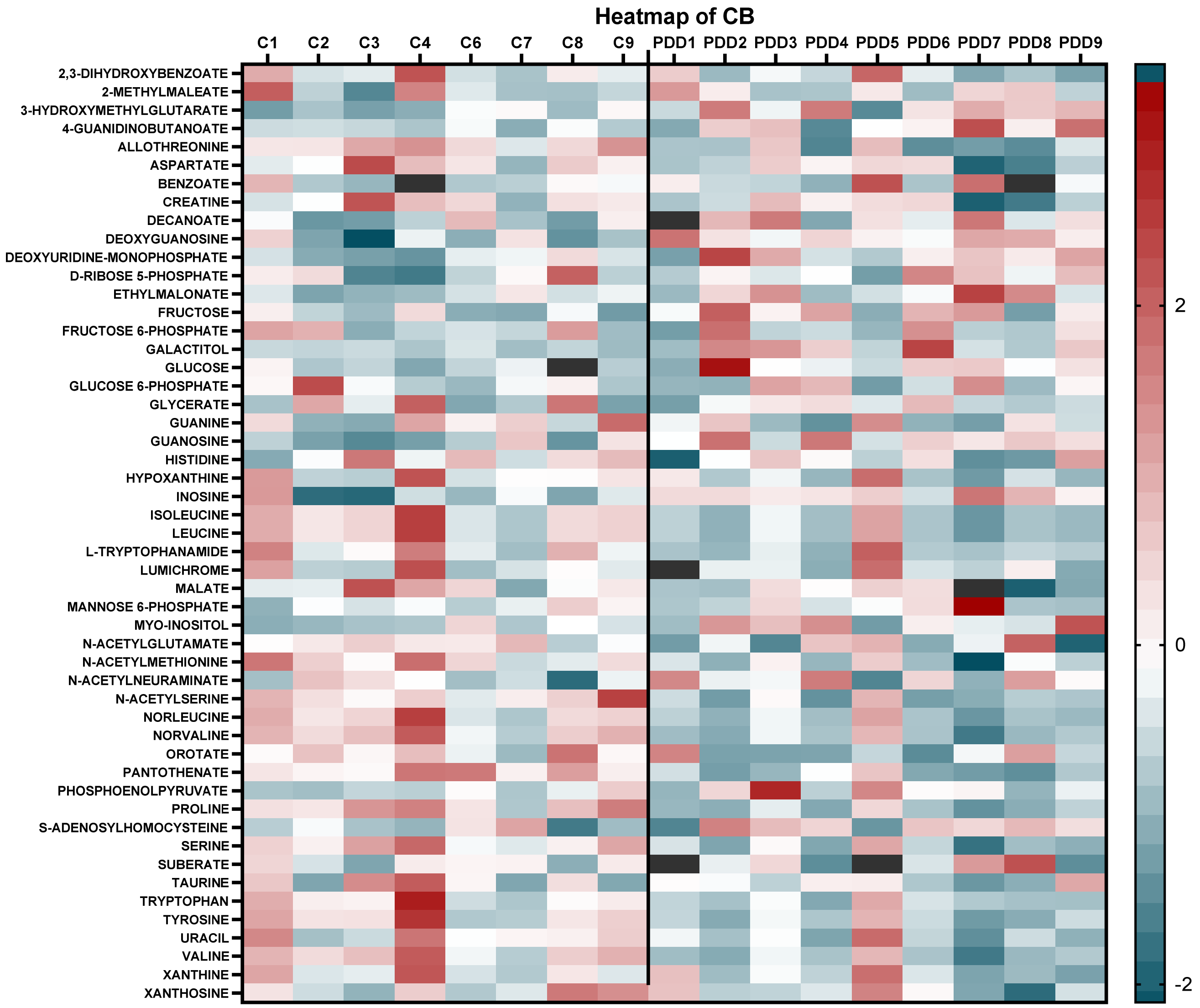


Heat map created using z-score normalised data in GraphPad Prism v9.1.2. All identified metabolites are shown. The darker the colour, the greater the difference in concentration relative to the mean; the red colour denotes increased relative concentrations; the blue colour denotes decreased relative concentrations. Black rectangles denote excluded values. C = control; PDD = Parkinson's disease dementia case.

Additional File Figure 2C – Heat Map: MCX

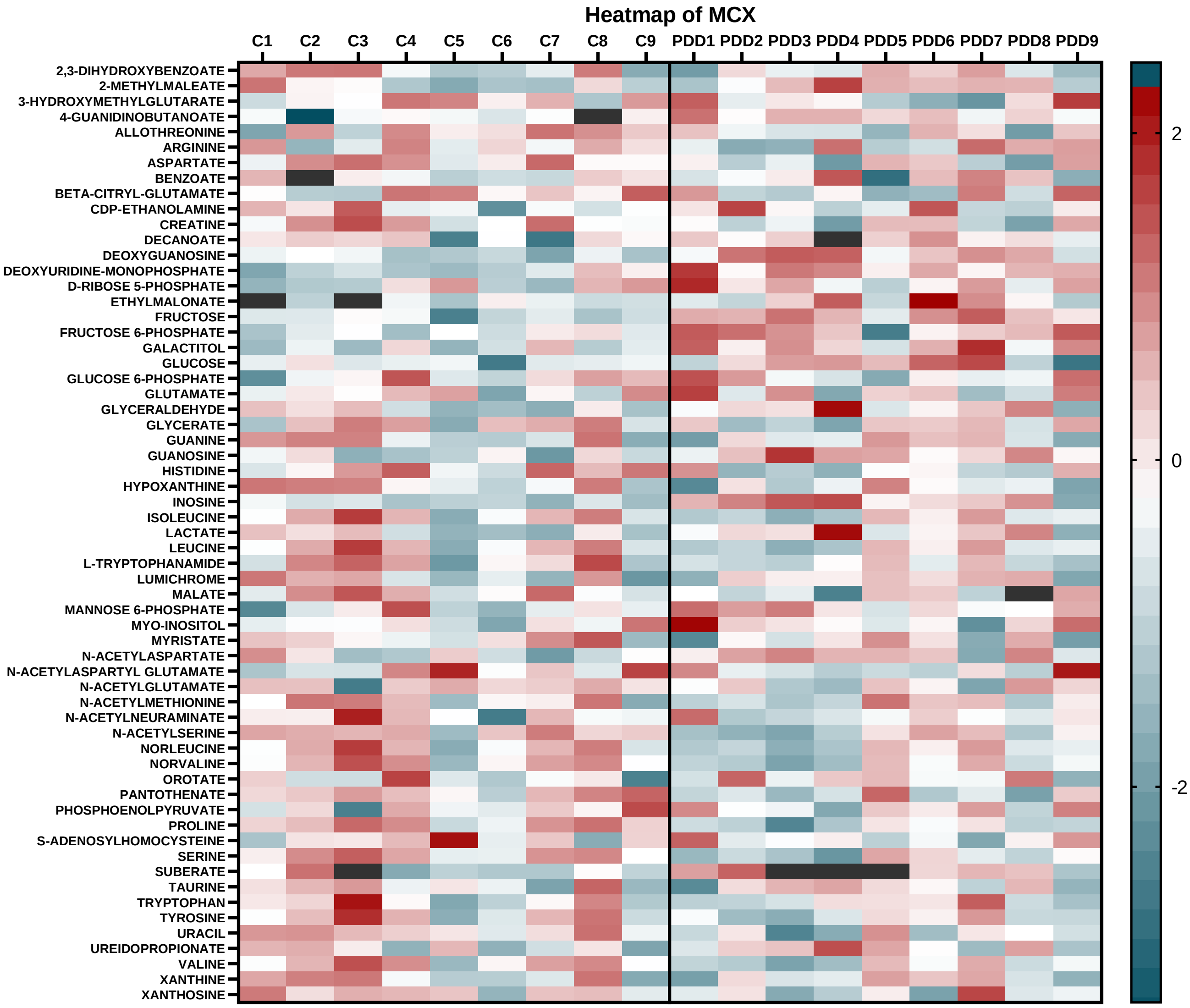


Heat map created using z-score normalised data in GraphPad Prism v9.1.2. All identified metabolites are shown. The darker the colour, the greater the difference in concentration relative to the mean; the red colour denotes increased relative concentrations; the blue colour denotes decreased relative concentrations. Black rectangles denote excluded values. C = control; PDD = Parkinson's disease dementia case.

# Additional File Figure 2D – Heat Map: MED

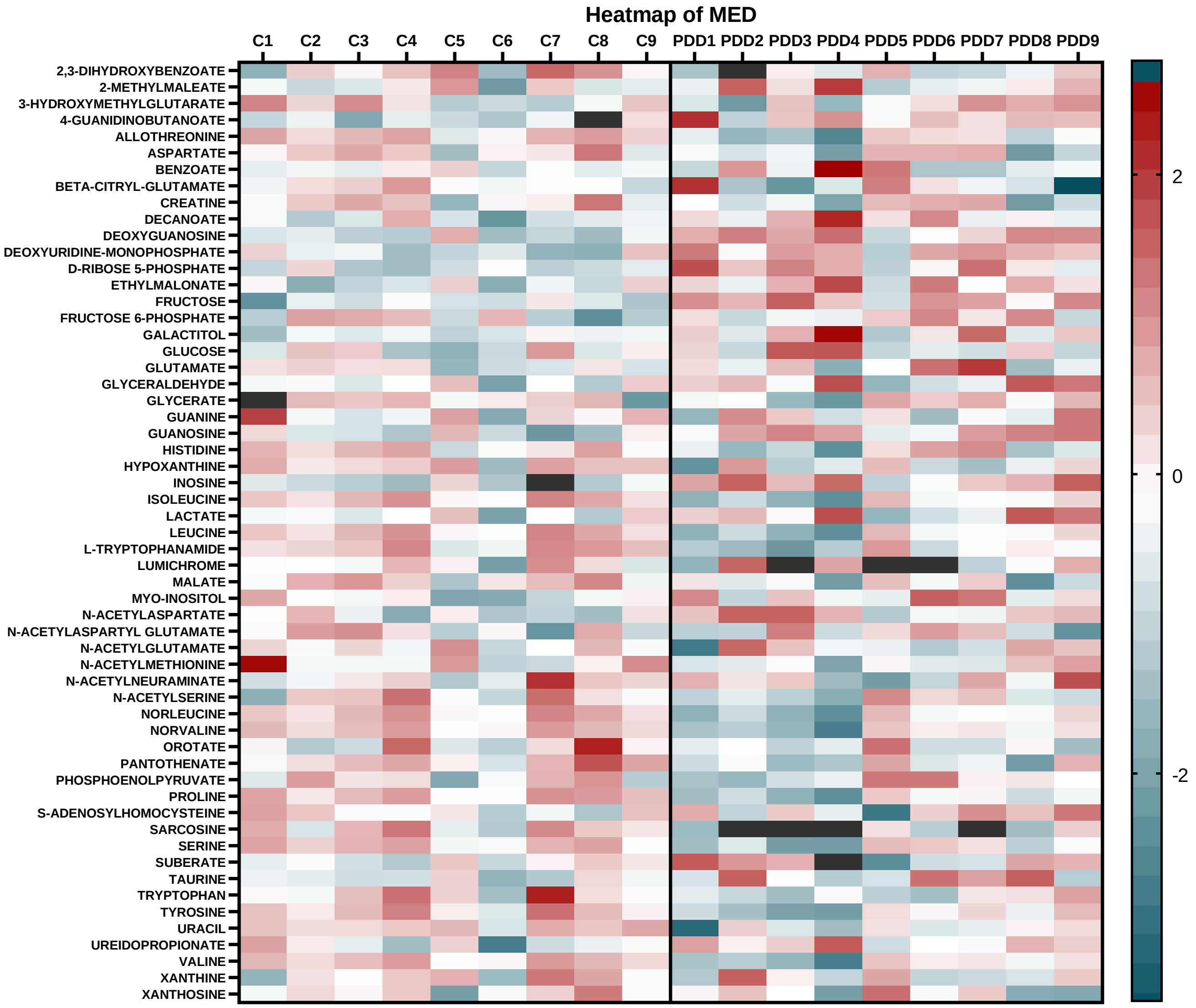


Heat map created using z-score normalised data in GraphPad Prism v9.1.2. All identified metabolites are shown. The darker the colour, the greater the difference in concentration relative to the mean; the red colour denotes increased relative concentrations; the blue colour denotes decreased relative concentrations. Black rectangles denote excluded values. C = control; PDD = Parkinson's disease dementia case.

Additional File Figure 2E – Heat Map: MTG

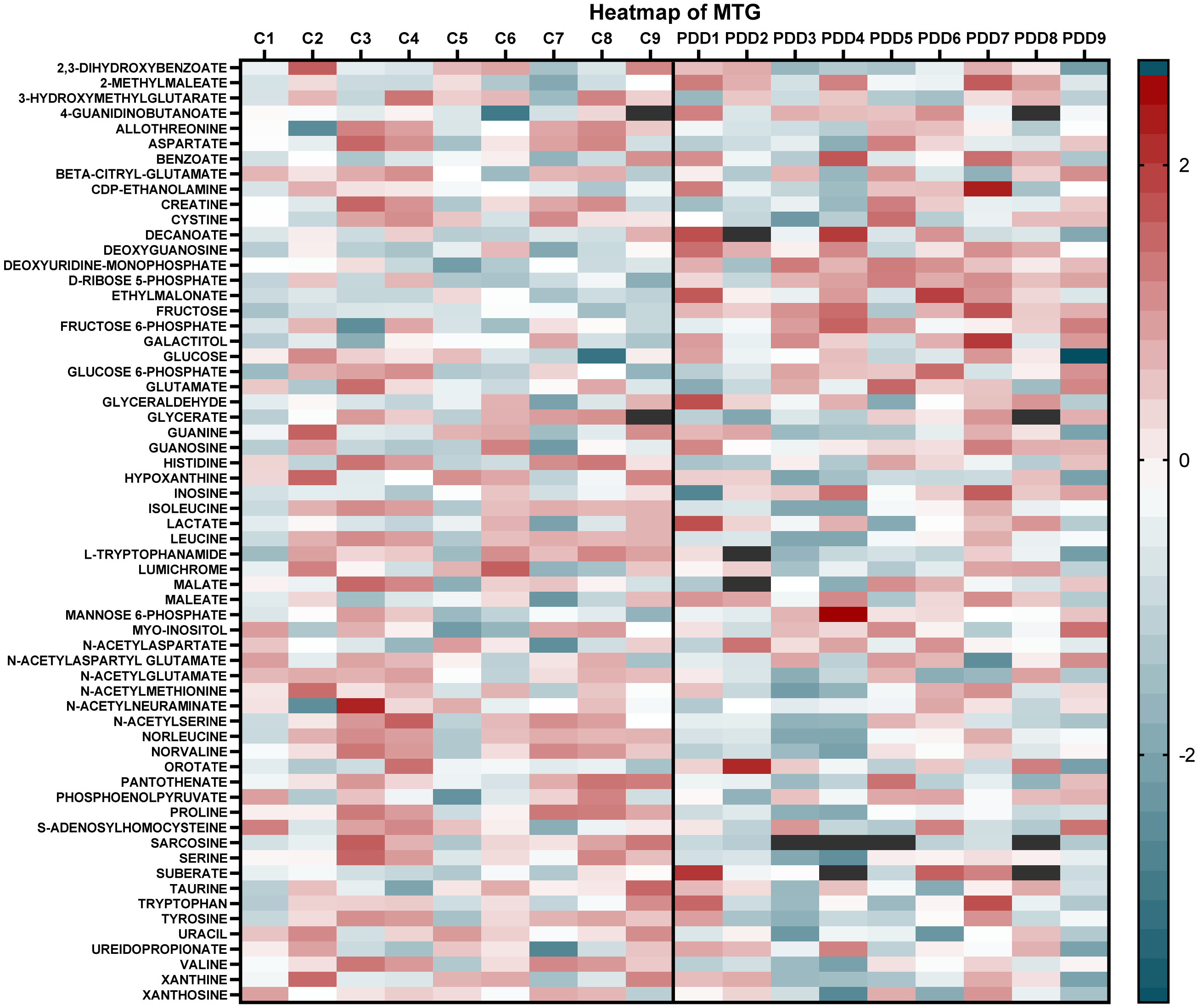


Heat map created using z-score normalised data in GraphPad Prism v9.1.2. All identified metabolites are shown. The darker the colour, the greater the difference in concentration relative to the mean; the red colour denotes increased relative concentrations; the blue colour denotes decreased relative concentrations. Black rectangles denote excluded values. C = control; PDD = Parkinson's disease dementia case.

Additional File Figure 2F – Heat Map: Pons

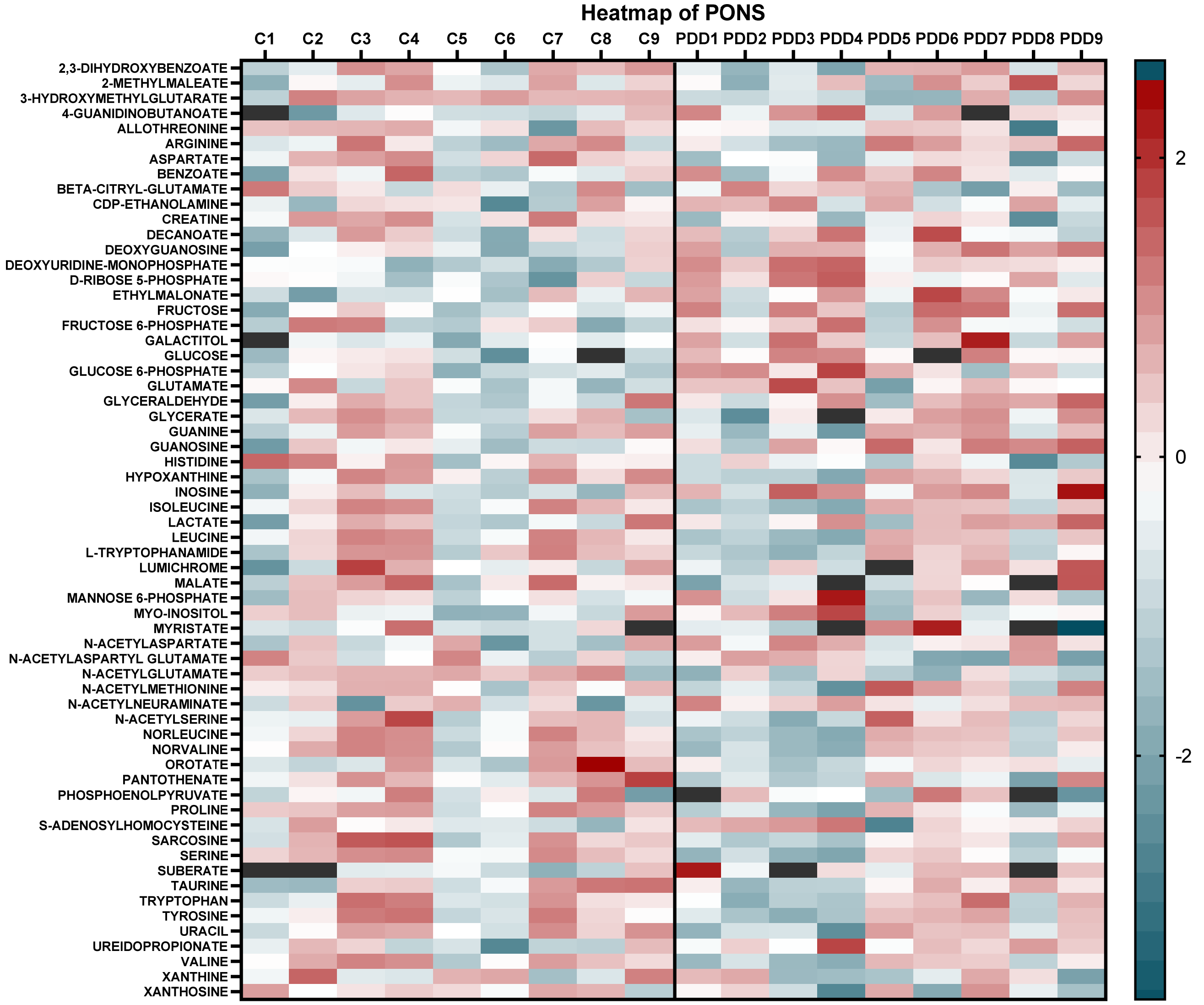


Heat map created using z-score normalised data in GraphPad Prism v9.1.2. All identified metabolites are shown. The darker the colour, the greater the difference in concentration relative to the mean; the red colour denotes increased relative concentrations; the blue colour denotes decreased relative concentrations. Black rectangles denote excluded values. C = control; PDD = Parkinson’s disease dementia case.

# Additional File Figure 2G – Heat Map: SN

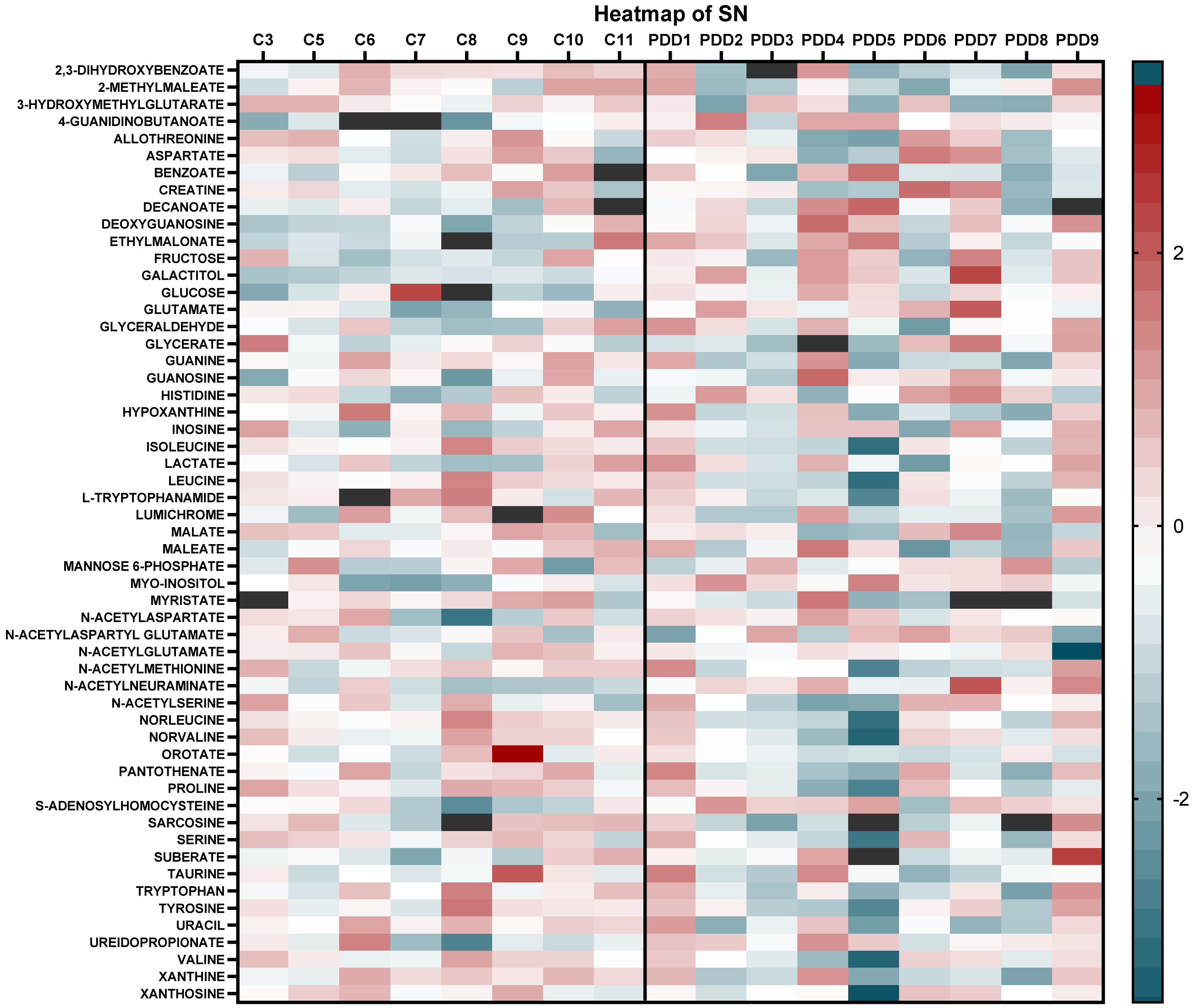


Heat map created using z-score normalised data in GraphPad Prism v9.1.2. All identified metabolites are shown. The darker the colour, the greater the difference in concentration relative to the mean; the red colour denotes increased relative concentrations; the blue colour denotes decreased relative concentrations. Black rectangles denote excluded values. C = control; PDD = Parkinson's disease dementia case.

Additional File Figure 2H – Heat Map: PVC

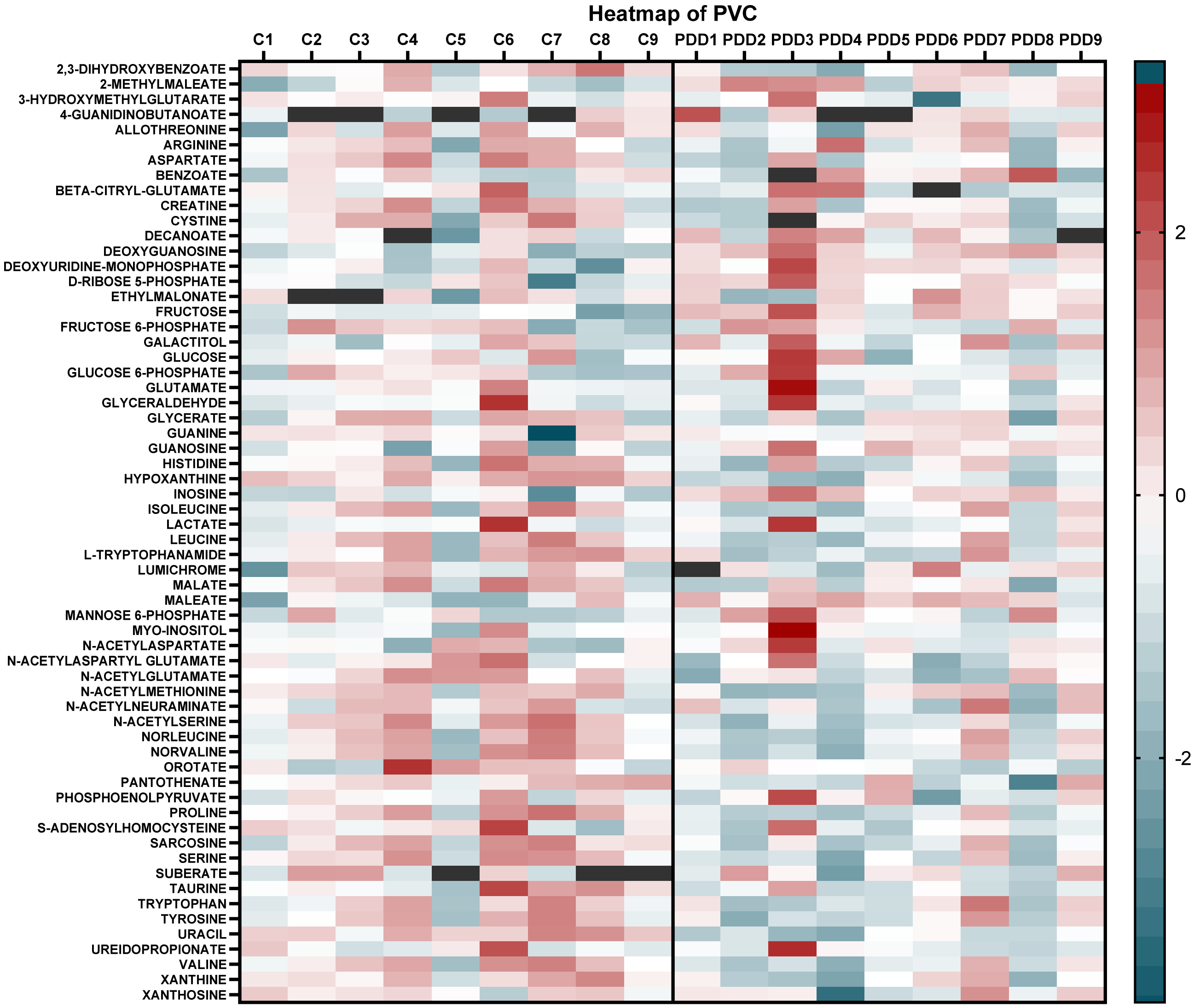


Heat map created using z-score normalised data in GraphPad Prism v9.1.2. All identified metabolites are shown. The darker the colour, the greater the difference in concentration relative to the mean; the red colour denotes increased relative concentrations; the blue colour denotes decreased relative concentrations. Black rectangles denote excluded values. C = control; PDD = Parkinson’s disease dementia case.

# Additional File Figure 3 – PCA Plots

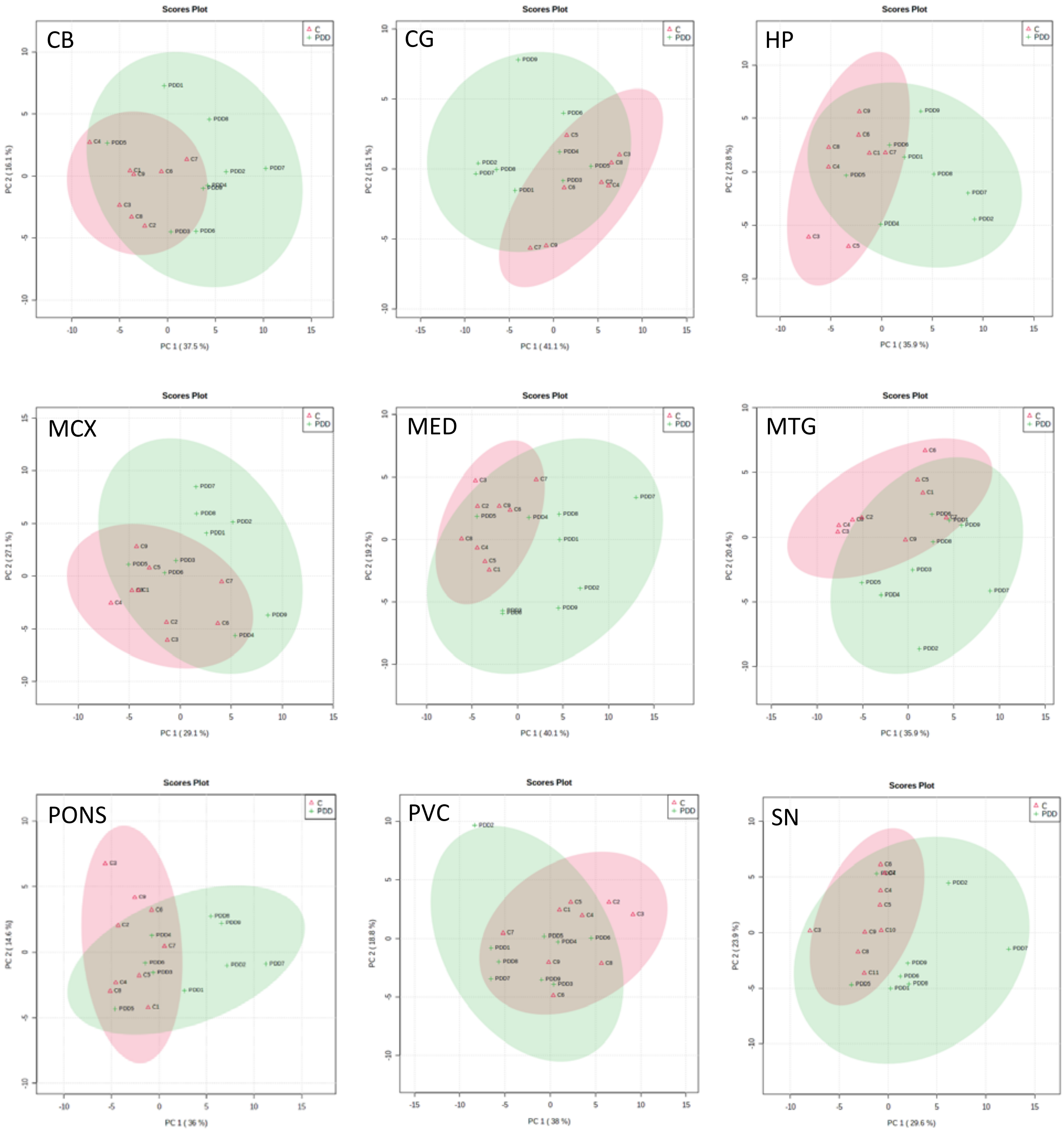


Two-dimensional PCA plots for human dry-weight post-mortem tissues from nine regions of PDD and control brains. Plots show 95% confidence intervals. No visible separation in apparent between cases and controls in any region. C = Control; PDD = Parkinson's disease dementia case.

# Additional File Figure 4A – Correlation Matrix: CG

| CG | | Amino Acids | | Glucose Pathways | | | | Purine Metabolism | | | | Other | |
|---|---|---|---|---|---|---|---|---|---|---|---|---|---|
| | | PROLINE | SERINE | D-RIBOSE 5-PHOSPHATE | FRUCTOSE | FRUCTOSE 6-PHOSPHATE | MANNOSE 6-PHOSPHATE | DEOXYGUANOSINE | DEOXYURIDINE-MONOPHOSPHATE | INOSINE | URACIL | ETHYLMALONATE | SUBERATE |
| Amino Acids | PROLINE | 1.00 | 0.93 | -0.58 | -0.55 | -0.73 | -0.64 | -0.60 | -0.56 | -0.39 | 0.68 | -0.42 | -0.69 |
| | SERINE | 0.93 | 1.00 | -0.59 | -0.49 | -0.65 | -0.54 | -0.63 | -0.63 | -0.40 | 0.68 | -0.40 | -0.73 |
| Glucose Pathways | D-RIBOSE 5-PHOSPHATE | -0.58 | -0.59 | 1.00 | 0.66 | 0.65 | 0.64 | 0.73 | 0.74 | 0.69 | -0.77 | 0.65 | 0.60 |
| | FRUCTOSE | -0.55 | -0.49 | 0.66 | 1.00 | 0.84 | 0.92 | 0.85 | 0.60 | 0.89 | -0.70 | 0.74 | 0.61 |
| | FRUCTOSE 6-PHOSPHATE | -0.73 | -0.65 | 0.65 | 0.84 | 1.00 | 0.90 | 0.68 | 0.63 | 0.69 | -0.71 | 0.62 | 0.51 |
| | MANNOSE 6-PHOSPHATE | -0.64 | -0.54 | 0.64 | 0.92 | 0.90 | 1.00 | 0.81 | 0.59 | 0.84 | -0.63 | 0.69 | 0.61 |
| Purine Metabolism | DEOXYGUANOSINE | -0.60 | -0.63 | 0.73 | 0.85 | 0.68 | 0.81 | 1.00 | 0.65 | 0.81 | -0.59 | 0.85 | 0.86 |
| | DEOXYURIDINE-MONOPHOSPHATE | -0.56 | -0.63 | 0.74 | 0.60 | 0.63 | 0.59 | 0.65 | 1.00 | 0.59 | -0.80 | 0.59 | 0.62 |
| | INOSINE | -0.39 | -0.40 | 0.69 | 0.89 | 0.69 | 0.84 | 0.81 | 0.59 | 1.00 | -0.63 | 0.65 | 0.57 |
| | URACIL | 0.68 | 0.68 | -0.77 | -0.70 | -0.71 | -0.63 | -0.59 | -0.80 | -0.63 | 1.00 | -0.50 | -0.50 |
| Other | ETHYLMALONATE | -0.42 | -0.40 | 0.65 | 0.74 | 0.62 | 0.69 | 0.85 | 0.59 | 0.65 | -0.50 | 1.00 | 0.80 |
| | SUBERATE | -0.69 | -0.73 | 0.60 | 0.61 | 0.51 | 0.61 | 0.86 | 0.62 | 0.57 | -0.50 | 0.80 | 1.00 |

Colour scale: 1.0, 0.5, 0, -0.5, -1.0

Matrix shows non-parametric Pearson correlation coefficient r values. Correlations were classed as non-significant (r -0.5–0.5; p > 0.05), weak (r = 0.5–06 or -0.5 to -0.6), moderate (r = 0.6–0.7 or -0.6 to -0.7), strong (r = 0.7–0.9 or -0.7 to -0.9), or very strong (r > 0.9 or < -0.9). All correlations of r = -0.5–0.5 had a p-value ≥ 0.05. The red colour denotes positive correlations while blue denotes negative correlations; the darker the colour, the stronger the correlation.

## Additional File Figure 4B – Correlation Matrix: CB

| CB | | Amino Acids | | | | | | | | | | Purine Metabolism | | | Other | | | |
|---|---|---|---|---|---|---|---|---|---|---|---|---|---|---|---|---|---|---|
| | | ALLOTHREONINE | ISOLEUCINE | LEUCINE | N-ACETYLSERINE | NORLEUCINE | NORVALINE | PROLINE | SERINE | TYROSINE | VALINE | DEOXYGUANOSINE | GUANOSINE | INOSINE | 3-HYDROXYMETHYLGLUTARATE | GALACTITOL | N-ACETYLMETHIONINE | PANTOTHENATE |
| Amino Acids | ALLOTHREONINE | 1.00 | 0.81 | 0.81 | 0.81 | 0.81 | 0.87 | 0.93 | 0.90 | 0.79 | 0.87 | -0.71 | -0.72 | -0.50 | -0.72 | -0.38 | 0.74 | 0.71 |
| | ISOLEUCINE | 0.81 | 1.00 | 1.00 | 0.90 | 1.00 | 0.93 | 0.88 | 0.97 | 0.90 | 0.93 | -0.51 | -0.68 | -0.30 | -0.89 | -0.46 | 0.88 | 0.74 |
| | LEUCINE | 0.81 | 1.00 | 1.00 | 0.90 | 1.00 | 0.93 | 0.88 | 0.97 | 0.90 | 0.93 | -0.51 | -0.68 | -0.30 | -0.89 | -0.46 | 0.88 | 0.74 |
| | N-ACETYLSERINE | 0.81 | 0.90 | 0.90 | 1.00 | 0.90 | 0.89 | 0.85 | 0.88 | 0.84 | 0.89 | -0.43 | -0.60 | -0.24 | -0.79 | -0.65 | 0.83 | 0.74 |
| | NORLEUCINE | 0.81 | 1.00 | 1.00 | 0.90 | 1.00 | 0.93 | 0.88 | 0.97 | 0.90 | 0.93 | -0.51 | -0.68 | -0.30 | -0.89 | -0.46 | 0.88 | 0.74 |
| | NORVALINE | 0.87 | 0.93 | 0.93 | 0.89 | 0.93 | 1.00 | 0.95 | 0.95 | 0.93 | 1.00 | -0.64 | -0.66 | -0.46 | -0.77 | -0.39 | 0.85 | 0.83 |
| | PROLINE | 0.93 | 0.88 | 0.88 | 0.85 | 0.88 | 0.95 | 1.00 | 0.94 | 0.88 | 0.95 | -0.75 | -0.71 | -0.57 | -0.74 | -0.37 | 0.77 | 0.78 |
| | SERINE | 0.90 | 0.97 | 0.97 | 0.88 | 0.97 | 0.95 | 0.94 | 1.00 | 0.91 | 0.95 | -0.65 | -0.74 | -0.50 | -0.86 | -0.49 | 0.85 | 0.75 |
| | TYROSINE | 0.79 | 0.90 | 0.90 | 0.84 | 0.90 | 0.93 | 0.88 | 0.91 | 1.00 | 0.93 | -0.51 | -0.64 | -0.36 | -0.81 | -0.36 | 0.80 | 0.67 |
| | VALINE | 0.87 | 0.93 | 0.93 | 0.89 | 0.93 | 1.00 | 0.95 | 0.95 | 0.93 | 1.00 | -0.64 | -0.66 | -0.46 | -0.77 | -0.39 | 0.85 | 0.83 |
| Purine Metabolism | DEOXYGUANOSINE | -0.71 | -0.51 | -0.51 | -0.43 | -0.51 | -0.64 | -0.75 | -0.65 | -0.51 | -0.64 | 1.00 | 0.64 | 0.89 | 0.43 | -0.03 | -0.44 | -0.54 |
| | GUANOSINE | -0.72 | -0.68 | -0.68 | -0.60 | -0.68 | -0.66 | -0.71 | -0.74 | -0.64 | -0.66 | 0.64 | 1.00 | 0.54 | 0.80 | 0.19 | -0.66 | -0.60 |
| | INOSINE | -0.50 | -0.30 | -0.30 | -0.24 | -0.30 | -0.46 | -0.57 | -0.50 | -0.36 | -0.46 | 0.89 | 0.54 | 1.00 | 0.29 | 0.03 | -0.26 | -0.47 |
| Other | 3-HYDROXYMETHYLGLUTARATE | -0.72 | -0.89 | -0.89 | -0.79 | -0.89 | -0.77 | -0.74 | -0.86 | -0.81 | -0.77 | 0.43 | 0.80 | 0.29 | 1.00 | 0.40 | -0.81 | -0.67 |
| | GALACTITOL | -0.38 | -0.46 | -0.46 | -0.65 | -0.46 | -0.39 | -0.37 | -0.49 | -0.36 | -0.39 | -0.03 | 0.19 | 0.03 | 0.40 | 1.00 | -0.45 | -0.42 |
| | N-ACETYLMETHIONINE | 0.74 | 0.88 | 0.88 | 0.83 | 0.88 | 0.85 | 0.77 | 0.85 | 0.80 | 0.85 | -0.44 | -0.66 | -0.26 | -0.81 | -0.45 | 1.00 | 0.71 |
| | PANTOTHENATE | 0.71 | 0.74 | 0.74 | 0.74 | 0.74 | 0.83 | 0.78 | 0.75 | 0.67 | 0.83 | -0.54 | -0.60 | -0.47 | -0.67 | -0.42 | 0.71 | 1.00 |

Colour scale: 1.0, 0.5, 0, -0.5, -1.0

Matrix shows non-parametric Pearson correlation coefficient r values. Correlations were classed as non-significant (r -0.5–0.5; p > 0.05), weak (r = 0.5–06 or -0.5 to -0.6), moderate (r = 0.6–0.7 or -0.6 to -0.7), strong (r = 0.7–0.9 or -0.7 to -0.9), or very strong (r > 0.9 or < -0.9). All correlations of r = -0.5–0.5 had a p-value ≥ 0.05. The red colour denotes positive correlations while blue denotes negative correlations; the darker the colour, the stronger the correlation.

## Additional File Figure 4C – Correlation Matrix: MCX

| MCX | | Amino Acids | | Glucose Pathways | Purine Metabolism | | | | Other |
|---|---|---|---|---|---|---|---|---|---|
| | | PROLINE | SERINE | FRUCTOSE | DEOXYGUANOSINE | DEOXYURIDINE-MONOPHOSPHATE | GUANOSINE | INOSINE | GALACTITOL |
| Amino Acids | PROLINE | 1.00 | 0.86 | -0.44 | -0.58 | -0.50 | -0.63 | -0.56 | -0.36 |
| | SERINE | 0.86 | 1.00 | -0.49 | -0.52 | -0.46 | -0.48 | -0.60 | -0.44 |
| Glucose Pathways | FRUCTOSE | -0.44 | -0.49 | 1.00 | 0.72 | 0.61 | 0.37 | 0.64 | 0.81 |
| Purine Metabolism | DEOXYGUANOSINE | -0.58 | -0.52 | 0.72 | 1.00 | 0.53 | 0.79 | 0.92 | 0.37 |
| | DEOXYURIDINE-MONOPHOSPHATE | -0.50 | -0.46 | 0.61 | 0.53 | 1.00 | 0.47 | 0.49 | 0.63 |
| | GUANOSINE | -0.63 | -0.48 | 0.37 | 0.79 | 0.47 | 1.00 | 0.70 | 0.22 |
| | INOSINE | -0.56 | -0.60 | 0.64 | 0.92 | 0.49 | 0.70 | 1.00 | 0.26 |
| Other | GALACTITOL | -0.36 | -0.44 | 0.81 | 0.37 | 0.63 | 0.22 | 0.26 | 1.00 |

Colour scale: 1.0, 0.5, 0, -0.5, -1.0

Matrix shows non-parametric Pearson correlation coefficient r values. Correlations were classed as non-significant (r -0.5–0.5; p > 0.05), weak (r = 0.5–06 or -0.5 to -0.6), moderate (r = 0.6–0.7 or -0.6 to -0.7), strong (r = 0.7–0.9 or -0.7 to -0.9), or very strong (r > 0.9 or < -0.9). All correlations of r = -0.5–0.5 had a p-value ≥ 0.05. The red colour denotes positive correlations while blue denotes negative correlations; the darker the colour, the stronger the correlation.

# Additional File Figure 4D – Correlation Matrix: MED

| | | Amino Acids | | | | | | | | | Glucose Pathways | | Purine Metabolism | | | | | | | Other | | | | | | |
|---|---|---|---|---|---|---|---|---|---|---|---|---|---|---|---|---|---|---|---|---|---|---|---|---|---|---|
| | **MED** | ALLOTHREONINE | HISTIDINE | ISOLEUCINE | LEUCINE | NORLEUCINE | NORVALINE | PROLINE | SERINE | VALINE | D-RIBOSE 5-PHOSPHATE | FRUCTOSE | DEOXYGUANOSINE | DEOXYURIDINE-MONOPHOSPHATE | GUANOSINE | HYPOXANTHINE | INOSINE | URACIL | UREIDOPROPIONATE | 4-GUANIDINOBUTANOATE | DECANOATE | ETHYLMALONATE | GALACTITOL | L-TRYPTOPHANAMIDE | N-ACETYLASPARTATE | PANTOTHENATE |
| Amino Acids | ALLOTHREONINE | 1.00 | 0.82 | 0.87 | 0.87 | 0.87 | 0.94 | 0.93 | 0.94 | 0.94 | -0.76 | -0.52 | -0.87 | -0.58 | -0.73 | 0.30 | -0.80 | 0.53 | -0.60 | -0.41 | -0.20 | -0.52 | -0.35 | 0.85 | -0.79 | 0.70 |
| | HISTIDINE | 0.82 | 1.00 | 0.57 | 0.57 | 0.57 | 0.70 | 0.70 | 0.83 | 0.70 | -0.40 | -0.26 | -0.71 | -0.22 | -0.53 | -0.04 | -0.64 | 0.20 | -0.54 | -0.29 | -0.08 | -0.41 | -0.10 | 0.57 | -0.71 | 0.45 |
| | ISOLEUCINE | 0.87 | 0.57 | 1.00 | 1.00 | 1.00 | 0.96 | 0.93 | 0.86 | 0.96 | -0.89 | -0.51 | -0.74 | -0.77 | -0.60 | 0.54 | -0.69 | 0.72 | -0.62 | -0.49 | -0.41 | -0.57 | -0.40 | 0.91 | -0.70 | 0.82 |
| | LEUCINE | 0.87 | 0.57 | 1.00 | 1.00 | 1.00 | 0.96 | 0.93 | 0.86 | 0.96 | -0.89 | -0.51 | -0.74 | -0.77 | -0.60 | 0.54 | -0.69 | 0.72 | -0.62 | -0.49 | -0.41 | -0.57 | -0.40 | 0.91 | -0.70 | 0.82 |
| | NORLEUCINE | 0.87 | 0.57 | 1.00 | 1.00 | 1.00 | 0.96 | 0.93 | 0.86 | 0.96 | -0.89 | -0.51 | -0.74 | -0.77 | -0.60 | 0.54 | -0.69 | 0.72 | -0.62 | -0.49 | -0.41 | -0.57 | -0.40 | 0.91 | -0.70 | 0.82 |
| | NORVALINE | 0.94 | 0.70 | 0.96 | 0.96 | 0.96 | 1.00 | 0.96 | 0.92 | 1.00 | -0.83 | -0.53 | -0.78 | -0.66 | -0.65 | 0.47 | -0.72 | 0.68 | -0.60 | -0.46 | -0.32 | -0.55 | -0.37 | 0.91 | -0.71 | 0.76 |
| | PROLINE | 0.93 | 0.70 | 0.93 | 0.93 | 0.93 | 0.96 | 1.00 | 0.90 | 0.96 | -0.81 | -0.62 | -0.82 | -0.72 | -0.72 | 0.51 | -0.80 | 0.73 | -0.65 | -0.59 | -0.42 | -0.59 | -0.43 | 0.89 | -0.75 | 0.76 |
| | SERINE | 0.94 | 0.83 | 0.86 | 0.86 | 0.86 | 0.92 | 0.90 | 1.00 | 0.92 | -0.78 | -0.48 | -0.84 | -0.65 | -0.70 | 0.34 | -0.78 | 0.49 | -0.66 | -0.57 | -0.26 | -0.61 | -0.41 | 0.79 | -0.80 | 0.67 |
| | VALINE | 0.94 | 0.70 | 0.96 | 0.96 | 0.96 | 1.00 | 0.96 | 0.92 | 1.00 | -0.83 | -0.53 | -0.78 | -0.66 | -0.65 | 0.47 | -0.72 | 0.68 | -0.60 | -0.46 | -0.32 | -0.55 | -0.37 | 0.91 | -0.71 | 0.76 |
| Glucose Pathways | D-RIBOSE 5-PHOSPHATE | -0.76 | -0.40 | -0.89 | -0.89 | -0.89 | -0.83 | -0.81 | -0.78 | -0.83 | 1.00 | 0.63 | 0.67 | 0.78 | 0.59 | -0.61 | 0.68 | -0.67 | 0.58 | 0.54 | 0.24 | 0.40 | 0.60 | -0.79 | 0.69 | -0.70 |
| | FRUCTOSE | -0.52 | -0.26 | -0.51 | -0.51 | -0.51 | -0.53 | -0.62 | -0.48 | -0.53 | 0.63 | 1.00 | 0.58 | 0.54 | 0.45 | -0.42 | 0.66 | -0.49 | 0.33 | 0.62 | 0.38 | 0.44 | 0.78 | -0.56 | 0.49 | -0.42 |
| Purine Metabolism | DEOXYGUANOSINE | -0.87 | -0.71 | -0.74 | -0.74 | -0.74 | -0.78 | -0.82 | -0.84 | -0.78 | 0.67 | 0.58 | 1.00 | 0.66 | 0.88 | -0.09 | 0.95 | -0.30 | 0.77 | 0.59 | 0.39 | 0.72 | 0.43 | -0.73 | 0.86 | -0.56 |
| | DEOXYURIDINE-MONOPHOSPHATE | -0.58 | -0.22 | -0.77 | -0.77 | -0.77 | -0.66 | -0.72 | -0.65 | -0.66 | 0.78 | 0.54 | 0.66 | 1.00 | 0.70 | -0.63 | 0.68 | -0.60 | 0.64 | 0.66 | 0.51 | 0.64 | 0.58 | -0.69 | 0.61 | -0.70 |
| | GUANOSINE | -0.73 | -0.53 | -0.60 | -0.60 | -0.60 | -0.65 | -0.72 | -0.70 | -0.65 | 0.59 | 0.45 | 0.88 | 0.70 | 1.00 | -0.16 | 0.87 | -0.26 | 0.72 | 0.50 | 0.33 | 0.65 | 0.33 | -0.63 | 0.73 | -0.51 |
| | HYPOXANTHINE | 0.30 | -0.04 | 0.54 | 0.54 | 0.54 | 0.47 | 0.51 | 0.34 | 0.47 | -0.61 | -0.42 | -0.09 | -0.63 | -0.16 | 1.00 | -0.10 | 0.86 | -0.15 | -0.45 | -0.23 | -0.16 | -0.54 | 0.40 | -0.11 | 0.61 |
| | INOSINE | -0.80 | -0.64 | -0.69 | -0.69 | -0.69 | -0.72 | -0.80 | -0.78 | -0.72 | 0.68 | 0.66 | 0.95 | 0.68 | 0.87 | -0.10 | 1.00 | -0.26 | 0.75 | 0.61 | 0.28 | 0.64 | 0.50 | -0.72 | 0.81 | -0.49 |
| | URACIL | 0.53 | 0.20 | 0.72 | 0.72 | 0.72 | 0.68 | 0.73 | 0.49 | 0.68 | -0.67 | -0.49 | -0.30 | -0.60 | -0.26 | 0.86 | -0.26 | 1.00 | -0.26 | -0.45 | -0.38 | -0.20 | -0.42 | 0.60 | -0.27 | 0.70 |
| | UREIDOPROPIONATE | -0.60 | -0.54 | -0.62 | -0.62 | -0.62 | -0.60 | -0.65 | -0.66 | -0.60 | 0.58 | 0.33 | 0.77 | 0.64 | 0.72 | -0.15 | 0.75 | -0.26 | 1.00 | 0.57 | 0.40 | 0.66 | 0.30 | -0.60 | 0.80 | -0.55 |
| Other | 4-GUANIDINOBUTANOATE | -0.41 | -0.29 | -0.49 | -0.49 | -0.49 | -0.46 | -0.59 | -0.57 | -0.46 | 0.54 | 0.62 | 0.59 | 0.66 | 0.50 | -0.45 | 0.61 | -0.45 | 0.57 | 1.00 | 0.58 | 0.71 | 0.70 | -0.30 | 0.43 | -0.36 |
| | DECANOATE | -0.20 | -0.08 | -0.41 | -0.41 | -0.41 | -0.32 | -0.42 | -0.26 | -0.32 | 0.24 | 0.38 | 0.39 | 0.51 | 0.33 | -0.23 | 0.28 | -0.38 | 0.40 | 0.58 | 1.00 | 0.64 | 0.34 | -0.28 | 0.23 | -0.31 |
| | ETHYLMALONATE | -0.52 | -0.41 | -0.57 | -0.57 | -0.57 | -0.55 | -0.59 | -0.61 | -0.55 | 0.40 | 0.44 | 0.72 | 0.64 | 0.65 | -0.16 | 0.64 | -0.20 | 0.66 | 0.71 | 0.64 | 1.00 | 0.43 | -0.51 | 0.49 | -0.53 |
| | GALACTITOL | -0.35 | -0.10 | -0.40 | -0.40 | -0.40 | -0.37 | -0.43 | -0.41 | -0.37 | 0.60 | 0.78 | 0.43 | 0.58 | 0.33 | -0.54 | 0.50 | -0.42 | 0.30 | 0.70 | 0.34 | 0.43 | 1.00 | -0.32 | 0.42 | -0.30 |
| | L-TRYPTOPHANAMIDE | 0.85 | 0.57 | 0.91 | 0.91 | 0.91 | 0.91 | 0.89 | 0.79 | 0.91 | -0.79 | -0.56 | -0.73 | -0.69 | -0.63 | 0.40 | -0.72 | 0.60 | -0.60 | -0.30 | -0.28 | -0.51 | -0.32 | 1.00 | -0.72 | 0.75 |
| | N-ACETYLASPARTATE | -0.79 | -0.71 | -0.70 | -0.70 | -0.70 | -0.71 | -0.75 | -0.80 | -0.71 | 0.69 | 0.49 | 0.86 | 0.61 | 0.73 | -0.11 | 0.81 | -0.27 | 0.80 | 0.43 | 0.23 | 0.49 | 0.42 | -0.72 | 1.00 | -0.51 |
| | PANTOTHENATE | 0.70 | 0.45 | 0.82 | 0.82 | 0.82 | 0.76 | 0.76 | 0.67 | 0.76 | -0.70 | -0.42 | -0.56 | -0.70 | -0.51 | 0.61 | -0.49 | 0.70 | -0.55 | -0.36 | -0.31 | -0.53 | -0.30 | 0.75 | -0.51 | 1.00 |

1.0 / 0.5 / 0 / -0.5 / -1.0

Matrix shows non-parametric Pearson correlation coefficient r values. Correlations were classed as non-significant (r -0.5–0.5; p > 0.05), weak (r = 0.5–06 or -0.5 to -0.6), moderate (r = 0.6–0.7 or -0.6 to -0.7), strong (r = 0.7–0.9 or -0.7 to -0.9), or very strong (r > 0.9 or < -0.9). All correlations of r = -0.5–0.5 had a p-value ≥ 0.05. The red colour denotes positive correlations while blue denotes negative correlations; the darker the colour, the stronger the correlation.

# Additional File Figure 4E – Correlation Matrix: MTG

| | MTG | Amino Acids | | | | | | | | | Glucose Pathways | | | Purine Metabolism | | | | Other | | | | | | |
|---|---|---|---|---|---|---|---|---|---|---|---|---|---|---|---|---|---|---|---|---|---|---|---|---|
| | | HISTIDINE | ISOLEUCINE | LEUCINE | N-ACETYLSERINE | NORLEUCINE | NORVALINE | PROLINE | SARCOSINE | VALINE | D-RIBOSE 5-PHOSPHATE | FRUCTOSE | FRUCTOSE 6-PHOSPHATE | DEOXYGUANOSINE | DEOXYURIDINE-MONOPHOSPHATE | HYPOXANTHINE | URACIL | 2-METHYLMALEATE | 4-GUANIDINOBUTANOATE | ETHYLMALONATE | GALACTITOL | L-TRYPTOPHANAMIDE | N-ACETYLGLUTAMATE | PANTOTHENATE |
| Amino Acids | HISTIDINE | 1.00 | 0.62 | 0.62 | 0.62 | 0.62 | 0.76 | 0.76 | 0.58 | 0.76 | -0.01 | -0.45 | -0.03 | -0.84 | 0.03 | -0.36 | -0.22 | -0.76 | -0.12 | -0.74 | -0.27 | 0.16 | 0.28 | 0.66 |
| | ISOLEUCINE | 0.62 | 1.00 | 1.00 | 0.79 | 1.00 | 0.94 | 0.81 | 0.72 | 0.94 | -0.10 | -0.24 | -0.20 | -0.40 | -0.25 | 0.08 | 0.11 | -0.41 | -0.39 | -0.50 | -0.16 | 0.67 | 0.38 | 0.50 |
| | LEUCINE | 0.62 | 1.00 | 1.00 | 0.79 | 1.00 | 0.94 | 0.81 | 0.72 | 0.94 | -0.10 | -0.24 | -0.20 | -0.40 | -0.25 | 0.08 | 0.11 | -0.41 | -0.39 | -0.50 | -0.16 | 0.67 | 0.38 | 0.50 |
| | N-ACETYLSERINE | 0.62 | 0.79 | 0.79 | 1.00 | 0.79 | 0.82 | 0.85 | 0.73 | 0.82 | -0.20 | -0.51 | -0.27 | -0.52 | -0.30 | 0.15 | 0.06 | -0.56 | -0.24 | -0.52 | -0.35 | 0.68 | 0.51 | 0.58 |
| | NORLEUCINE | 0.62 | 1.00 | 1.00 | 0.79 | 1.00 | 0.94 | 0.81 | 0.72 | 0.94 | -0.10 | -0.24 | -0.20 | -0.40 | -0.25 | 0.08 | 0.11 | -0.41 | -0.39 | -0.50 | -0.16 | 0.67 | 0.38 | 0.50 |
| | NORVALINE | 0.76 | 0.94 | 0.94 | 0.82 | 0.94 | 1.00 | 0.92 | 0.84 | 1.00 | -0.21 | -0.43 | -0.26 | -0.56 | -0.28 | 0.05 | 0.12 | -0.56 | -0.42 | -0.66 | -0.30 | 0.70 | 0.43 | 0.66 |
| | PROLINE | 0.76 | 0.81 | 0.81 | 0.85 | 0.81 | 0.92 | 1.00 | 0.87 | 0.92 | -0.39 | -0.64 | -0.36 | -0.69 | -0.37 | 0.15 | 0.22 | -0.63 | -0.38 | -0.77 | -0.45 | 0.63 | 0.50 | 0.72 |
| | SARCOSINE | 0.58 | 0.72 | 0.72 | 0.73 | 0.72 | 0.84 | 0.87 | 1.00 | 0.84 | -0.32 | -0.53 | -0.26 | -0.39 | -0.12 | 0.10 | 0.22 | -0.53 | 0.01 | -0.68 | -0.55 | 0.69 | 0.64 | 0.60 |
| | VALINE | 0.76 | 0.94 | 0.94 | 0.82 | 0.94 | 1.00 | 0.92 | 0.84 | 1.00 | -0.21 | -0.43 | -0.26 | -0.56 | -0.28 | 0.05 | 0.12 | -0.56 | -0.42 | -0.66 | -0.30 | 0.70 | 0.43 | 0.66 |
| Glucose Pathways | D-RIBOSE 5-PHOSPHATE | -0.01 | -0.10 | -0.10 | -0.20 | -0.10 | -0.21 | -0.39 | -0.32 | -0.21 | 1.00 | 0.52 | 0.81 | 0.24 | 0.70 | -0.65 | -0.54 | 0.32 | 0.57 | 0.37 | 0.50 | -0.38 | -0.21 | -0.17 |
| | FRUCTOSE | -0.45 | -0.24 | -0.24 | -0.51 | -0.24 | -0.43 | -0.64 | -0.53 | -0.43 | 0.52 | 1.00 | 0.35 | 0.64 | 0.46 | -0.52 | -0.54 | 0.37 | 0.15 | 0.64 | 0.81 | -0.36 | -0.69 | -0.70 |
| | FRUCTOSE 6-PHOSPHATE | -0.03 | -0.20 | -0.20 | -0.27 | -0.20 | -0.26 | -0.36 | -0.26 | -0.26 | 0.81 | 0.35 | 1.00 | 0.06 | 0.44 | -0.55 | -0.27 | 0.16 | 0.36 | 0.06 | 0.42 | -0.33 | -0.03 | -0.08 |
| Purine Metabolism | DEOXYGUANOSINE | -0.84 | -0.40 | -0.40 | -0.52 | -0.40 | -0.56 | -0.69 | -0.39 | -0.56 | 0.24 | 0.64 | 0.06 | 1.00 | 0.22 | 0.12 | -0.07 | 0.76 | 0.24 | 0.85 | 0.37 | -0.03 | -0.42 | -0.66 |
| | DEOXYURIDINE-MONOPHOSPHATE | 0.03 | -0.25 | -0.25 | -0.30 | -0.25 | -0.28 | -0.37 | -0.12 | -0.28 | 0.70 | 0.46 | 0.44 | 0.22 | 1.00 | -0.64 | -0.74 | 0.21 | 0.72 | 0.30 | 0.35 | -0.57 | -0.36 | -0.17 |
| | HYPOXANTHINE | -0.36 | 0.08 | 0.08 | 0.15 | 0.08 | 0.05 | 0.15 | 0.10 | 0.05 | -0.65 | -0.52 | -0.55 | 0.12 | -0.64 | 1.00 | 0.83 | 0.20 | -0.28 | -0.02 | -0.53 | 0.49 | 0.48 | 0.06 |
| | URACIL | -0.22 | 0.11 | 0.11 | 0.06 | 0.11 | 0.12 | 0.22 | 0.22 | 0.12 | -0.54 | -0.54 | -0.27 | -0.07 | -0.74 | 0.83 | 1.00 | 0.12 | -0.47 | -0.23 | -0.49 | 0.47 | 0.54 | 0.10 |
| Other | 2-METHYLMALEATE | -0.76 | -0.41 | -0.41 | -0.56 | -0.41 | -0.56 | -0.63 | -0.53 | -0.56 | 0.32 | 0.37 | 0.16 | 0.76 | 0.21 | 0.20 | 0.12 | 1.00 | 0.32 | 0.69 | 0.15 | -0.21 | -0.14 | -0.36 |
| | 4-GUANIDINOBUTANOATE | -0.12 | -0.39 | -0.39 | -0.24 | -0.39 | -0.42 | -0.38 | 0.01 | -0.42 | 0.57 | 0.15 | 0.36 | 0.24 | 0.72 | -0.28 | -0.47 | 0.32 | 1.00 | 0.31 | 0.18 | -0.32 | -0.07 | -0.17 |
| | ETHYLMALONATE | -0.74 | -0.50 | -0.50 | -0.52 | -0.50 | -0.66 | -0.77 | -0.68 | -0.66 | 0.37 | 0.64 | 0.06 | 0.85 | 0.30 | -0.02 | -0.23 | 0.69 | 0.31 | 1.00 | 0.47 | -0.31 | -0.51 | -0.75 |
| | GALACTITOL | -0.27 | -0.16 | -0.16 | -0.35 | -0.16 | -0.30 | -0.45 | -0.55 | -0.30 | 0.50 | 0.81 | 0.42 | 0.37 | 0.35 | -0.53 | -0.49 | 0.15 | 0.18 | 0.47 | 1.00 | -0.28 | -0.68 | -0.47 |
| | L-TRYPTOPHANAMIDE | 0.16 | 0.67 | 0.67 | 0.68 | 0.67 | 0.70 | 0.63 | 0.69 | 0.70 | -0.38 | -0.36 | -0.33 | -0.03 | -0.57 | 0.49 | 0.47 | -0.21 | -0.32 | -0.31 | -0.28 | 1.00 | 0.46 | 0.37 |
| | N-ACETYLGLUTAMATE | 0.28 | 0.38 | 0.38 | 0.51 | 0.38 | 0.43 | 0.50 | 0.64 | 0.43 | -0.21 | -0.69 | -0.03 | -0.42 | -0.36 | 0.48 | 0.54 | -0.14 | -0.07 | -0.51 | -0.68 | 0.46 | 1.00 | 0.50 |
| | PANTOTHENATE | 0.66 | 0.50 | 0.50 | 0.58 | 0.50 | 0.66 | 0.72 | 0.60 | 0.66 | -0.17 | -0.70 | -0.08 | -0.66 | -0.17 | 0.06 | 0.10 | -0.36 | -0.17 | -0.75 | -0.47 | 0.37 | 0.50 | 1.00 |

1.0 0.5 0 -0.5 -1.0

Matrix shows non-parametric Pearson correlation coefficient r values. Correlations were classed as non-significant (r -0.5–0.5; p > 0.05), weak (r = 0.5–06 or -0.5 to -0.6), moderate (r = 0.6–0.7 or -0.6 to -0.7), strong (r = 0.7–0.9 or -0.7 to -0.9), or very strong (r > 0.9 or < -0.9). All correlations of r = -0.5–0.5 had a p-value ≥ 0.05. The red colour denotes positive correlations while blue denotes negative correlations; the darker the colour, the stronger the correlation.

# Additional File Figure 4F – Correlation Matrix: Pons

| Pons | | Amino Acids | | | | | | | Glucose Pathways | | | | Purine Metabolism | | | | | Other | | | | | | | |
|---|---|---|---|---|---|---|---|---|---|---|---|---|---|---|---|---|---|---|---|---|---|---|---|---|---|
| | | ASPARTATE | CREATINE | NORVALINE | PANTOTHENATE | PROLINE | SERINE | VALINE | D-RIBOSE 5-PHOSPHATE | FRUCTOSE | GLUCOSE | GLUCOSE 6-PHOSPHATE | DEOXYGUANOSINE | DEOXYURIDINE-MONOPHOSPHATE | GUANOSINE | INOSINE | UREIDOPROPIONATE | 3-HYDROXYMETHYLGLUTARATE | 4-GUANIDINOBUTANOATE | ETHYLMALONATE | GALACTITOL | N-ACETYLASPARTATE | N-ACETYLGLUTAMATE | N-ACETYLNEURAMINATE | SUBERATE |
| Amino Acids | ASPARTATE | 1.00 | 0.99 | 0.82 | 0.47 | 0.81 | 0.81 | 0.82 | -0.65 | 0.02 | -0.15 | -0.27 | -0.40 | -0.55 | -0.36 | -0.28 | -0.40 | 0.49 | -0.58 | -0.32 | -0.03 | -0.58 | 0.51 | -0.56 | -0.66 |
| | CREATINE | 0.99 | 1.00 | 0.81 | 0.42 | 0.79 | 0.81 | 0.81 | -0.64 | 0.10 | -0.13 | -0.26 | -0.34 | -0.51 | -0.30 | -0.21 | -0.34 | 0.45 | -0.53 | -0.26 | 0.04 | -0.51 | 0.44 | -0.51 | -0.58 |
| | NORVALINE | 0.82 | 0.81 | 1.00 | 0.68 | 0.92 | 0.93 | 1.00 | -0.64 | 0.03 | -0.08 | -0.16 | -0.21 | -0.64 | | -0.15 | -0.21 | 0.38 | -0.59 | -0.27 | -0.16 | -0.58 | 0.40 | -0.49 | -0.51 |
| | PANTOTHENATE | 0.47 | 0.42 | 0.68 | 1.00 | 0.73 | 0.67 | 0.68 | -0.59 | -0.15 | -0.25 | -0.41 | -0.23 | -0.56 | -0.08 | [illegible] | -0.19 | 0.49 | -0.42 | -0.17 | -0.14 | -0.43 | 0.35 | -0.39 | -0.35 |
| | PROLINE | 0.81 | 0.79 | 0.92 | 0.73 | 1.00 | 0.94 | 0.92 | -0.61 | -0.21 | -0.29 | -0.25 | -0.45 | -0.73 | -0.26 | -0.41 | -0.43 | 0.29 | -0.61 | -0.33 | -0.20 | -0.70 | 0.49 | -0.58 | -0.66 |
| | SERINE | 0.81 | 0.81 | 0.93 | 0.67 | 0.94 | 1.00 | 0.93 | -0.66 | -0.07 | -0.25 | -0.28 | -0.34 | -0.70 | -0.18 | -0.31 | -0.30 | 0.30 | -0.61 | -0.27 | -0.14 | -0.59 | 0.50 | -0.47 | -0.51 |
| | VALINE | 0.82 | 0.81 | 1.00 | 0.68 | 0.92 | 0.93 | 1.00 | -0.64 | 0.03 | -0.08 | -0.16 | -0.21 | -0.64 | | -0.15 | -0.21 | 0.38 | -0.59 | -0.27 | -0.16 | -0.58 | 0.40 | -0.49 | -0.51 |
| Glucose Pathways | D-RIBOSE 5-PHOSPHATE | -0.65 | -0.64 | -0.64 | -0.59 | -0.61 | -0.66 | -0.64 | 1.00 | 0.01 | 0.44 | 0.50 | 0.24 | 0.62 | 0.20 | [illegible] | 0.31 | -0.50 | 0.51 | 0.12 | 0.07 | 0.42 | -0.29 | 0.28 | 0.34 |
| | FRUCTOSE | 0.02 | 0.10 | 0.03 | -0.15 | -0.21 | -0.07 | 0.03 | 0.01 | 1.00 | 0.81 | 0.16 | 0.80 | 0.47 | 0.58 | 0.87 | 0.40 | 0.26 | 0.57 | 0.62 | 0.88 | 0.37 | -0.45 | 0.29 | 0.54 |
| | GLUCOSE | -0.15 | -0.13 | -0.08 | -0.25 | -0.29 | -0.25 | -0.08 | 0.44 | 0.81 | 1.00 | 0.40 | 0.75 | 0.46 | 0.64 | 0.78 | 0.31 | 0.09 | 0.57 | 0.49 | 0.63 | 0.39 | -0.30 | 0.43 | 0.44 |
| | GLUCOSE 6-PHOSPHATE | -0.27 | -0.26 | -0.16 | -0.41 | -0.25 | -0.28 | -0.16 | 0.50 | 0.16 | 0.40 | 1.00 | 0.17 | 0.42 | 0.22 | 0.20 | 0.36 | -0.47 | 0.49 | 0.02 | -0.11 | 0.16 | -0.30 | 0.27 | 0.05 |
| Purine Metabolism | DEOXYGUANOSINE | -0.40 | -0.34 | -0.21 | -0.23 | -0.45 | -0.34 | -0.21 | 0.24 | 0.80 | 0.75 | 0.17 | 1.00 | 0.53 | 0.82 | 0.87 | 0.52 | 0.07 | 0.75 | 0.68 | 0.65 | 0.68 | -0.62 | 0.51 | 0.82 |
| | DEOXYURIDINE-MONOPHOSPHATE | -0.55 | -0.51 | -0.64 | -0.56 | -0.73 | -0.70 | -0.64 | 0.62 | 0.47 | 0.46 | 0.42 | 0.53 | 1.00 | 0.29 | 0.63 | 0.66 | -0.33 | 0.79 | 0.46 | 0.48 | 0.60 | -0.75 | 0.24 | 0.73 |
| | GUANOSINE | -0.36 | -0.30 | | -0.08 | -0.26 | -0.18 | | 0.20 | 0.58 | 0.64 | 0.22 | 0.82 | 0.29 | 1.00 | 0.76 | 0.47 | 0.01 | 0.35 | 0.41 | 0.39 | 0.56 | -0.56 | 0.51 | 0.64 |
| | INOSINE | -0.28 | -0.21 | -0.15 | [illegible] | -0.41 | -0.31 | -0.15 | [illegible] | 0.87 | 0.78 | 0.20 | 0.87 | 0.63 | 0.76 | 1.00 | 0.59 | 0.12 | 0.66 | 0.66 | 0.77 | 0.59 | -0.66 | 0.41 | 0.75 |
| | UREIDOPROPIONATE | -0.40 | -0.34 | -0.21 | -0.19 | -0.43 | -0.30 | -0.21 | 0.31 | 0.40 | 0.31 | 0.36 | 0.52 | 0.66 | 0.47 | 0.59 | 1.00 | -0.14 | 0.44 | 0.30 | 0.15 | 0.58 | -0.48 | 0.29 | 0.62 |
| Other | 3-HYDROXYMETHYLGLUTARATE | 0.49 | 0.45 | 0.38 | 0.49 | 0.29 | 0.30 | 0.38 | -0.50 | 0.26 | 0.09 | -0.47 | 0.07 | -0.33 | 0.01 | 0.12 | -0.14 | 1.00 | -0.43 | -0.25 | 0.22 | -0.16 | 0.38 | -0.05 | -0.18 |
| | 4-GUANIDINOBUTANOATE | -0.58 | -0.53 | -0.59 | -0.42 | -0.61 | -0.61 | -0.59 | 0.51 | 0.57 | 0.57 | 0.49 | 0.75 | 0.79 | 0.35 | 0.66 | 0.44 | -0.43 | 1.00 | 0.68 | 0.55 | 0.60 | -0.69 | 0.32 | 0.79 |
| | ETHYLMALONATE | -0.32 | -0.26 | -0.27 | -0.17 | -0.33 | -0.27 | -0.27 | 0.12 | 0.62 | 0.49 | 0.02 | 0.68 | 0.46 | 0.41 | 0.66 | 0.30 | -0.25 | 0.68 | 1.00 | 0.63 | 0.47 | -0.53 | 0.35 | 0.71 |
| | GALACTITOL | -0.03 | 0.04 | -0.16 | -0.14 | -0.20 | -0.14 | -0.16 | 0.07 | 0.88 | 0.63 | -0.11 | 0.65 | 0.48 | 0.39 | 0.77 | 0.15 | 0.22 | 0.55 | 0.63 | 1.00 | 0.30 | -0.47 | 0.22 | 0.57 |
| | N-ACETYLASPARTATE | -0.58 | -0.51 | -0.58 | -0.43 | -0.70 | -0.59 | -0.58 | 0.42 | 0.37 | 0.39 | 0.16 | 0.68 | 0.60 | 0.56 | 0.59 | 0.58 | -0.16 | 0.60 | 0.47 | 0.30 | 1.00 | -0.55 | 0.71 | 0.84 |
| | N-ACETYLGLUTAMATE | 0.51 | 0.44 | 0.40 | 0.35 | 0.49 | 0.50 | 0.40 | -0.29 | -0.45 | -0.30 | -0.30 | -0.62 | -0.75 | -0.56 | -0.66 | -0.48 | 0.38 | -0.69 | -0.53 | -0.47 | -0.55 | 1.00 | -0.23 | -0.79 |
| | N-ACETYLNEURAMINATE | -0.56 | -0.51 | -0.49 | -0.39 | -0.58 | -0.47 | -0.49 | 0.28 | 0.29 | 0.43 | 0.27 | 0.51 | 0.24 | 0.51 | 0.41 | 0.29 | -0.05 | 0.32 | 0.35 | 0.22 | 0.71 | -0.23 | 1.00 | 0.44 |
| | SUBERATE | -0.66 | -0.58 | -0.51 | -0.35 | -0.66 | -0.51 | -0.51 | 0.34 | 0.54 | 0.44 | 0.05 | 0.82 | 0.73 | 0.64 | 0.75 | 0.62 | -0.18 | 0.79 | 0.71 | 0.57 | 0.84 | -0.79 | 0.44 | 1.00 |

Colour scale: 1.0, 0.5, 0, -0.5, -1.0

Matrix shows non-parametric Pearson correlation coefficient r values. Correlations were classed as non-significant (r -0.5–0.5; p > 0.05), weak (r = 0.5–06 or -0.5 to -0.6), moderate (r = 0.6–0.7 or -0.6 to -0.7), strong (r = 0.7–0.9 or -0.7 to -0.9), or very strong (r > 0.9 or < -0.9). All correlations of r = -0.5–0.5 had a p-value ≥ 0.05. The red colour denotes positive correlations while blue denotes negative correlations; the darker the colour, the stronger the correlation.

## Additional File Figure 4G – Correlation Matrix: SN

| SN | | GLUTAMATE | HISTIDINE | ISOLEUCINE | LEUCINE | NORLEUCINE | PROLINE | SERINE | DEOXYGUANOSINE | GUANINE | HYPOXANTHINE | URACIL | XANTHINE | 2,3-DIHYDROXYBENZOATE | 4-GUANIDINOBUTANOATE | GALACTITOL | L-TRYPTOPHANAMIDE | MYO-INOSITOL | N-ACETYLMETHIONINE | N-ACETYLNEURAMINATE | S-ADENOSYLHOMOCYSTEINE |
|---|---|---|---|---|---|---|---|---|---|---|---|---|---|---|---|---|---|---|---|---|---|
| | | Amino Acids | | | | | | | Purine Metabolism | | | | | Other | | | | | | | |
| Amino Acids | GLUTAMATE | 1.00 | 0.82 | -0.34 | -0.34 | -0.34 | 0.07 | 0.08 | 0.03 | -0.62 | -0.67 | -0.68 | -0.63 | -0.61 | 0.25 | 0.20 | -0.46 | 0.84 | -0.60 | 0.26 | 0.40 |
| | HISTIDINE | 0.82 | 1.00 | -0.21 | -0.21 | -0.21 | 0.25 | 0.18 | -0.20 | -0.69 | -0.69 | -0.67 | -0.68 | -0.68 | 0.13 | 0.02 | -0.30 | 0.65 | -0.55 | 0.10 | 0.22 |
| | ISOLEUCINE | -0.34 | -0.21 | 1.00 | 1.00 | 1.00 | 0.71 | 0.78 | -0.29 | 0.48 | 0.57 | 0.57 | 0.47 | 0.38 | -0.59 | -0.32 | 0.66 | -0.54 | 0.73 | -0.43 | -0.78 |
| | LEUCINE | -0.34 | -0.21 | 1.00 | 1.00 | 1.00 | 0.71 | 0.78 | -0.29 | 0.48 | 0.57 | 0.57 | 0.47 | 0.38 | -0.59 | -0.32 | 0.66 | -0.54 | 0.73 | -0.43 | -0.78 |
| | NORLEUCINE | -0.34 | -0.21 | 1.00 | 1.00 | 1.00 | 0.71 | 0.78 | -0.29 | 0.48 | 0.57 | 0.57 | 0.47 | 0.38 | -0.59 | -0.32 | 0.66 | -0.54 | 0.73 | -0.43 | -0.78 |
| | PROLINE | 0.07 | 0.25 | 0.71 | 0.71 | 0.71 | 1.00 | 0.88 | -0.69 | 0.19 | 0.35 | 0.37 | 0.19 | 0.14 | -0.66 | -0.61 | 0.57 | -0.25 | 0.36 | -0.47 | -0.67 |
| | SERINE | 0.08 | 0.18 | 0.78 | 0.78 | 0.78 | 0.88 | 1.00 | -0.53 | 0.27 | 0.40 | 0.40 | 0.28 | 0.20 | -0.58 | -0.47 | 0.54 | -0.25 | 0.36 | -0.32 | -0.71 |
| Purine Metabolism | DEOXYGUANOSINE | 0.03 | -0.20 | -0.29 | -0.29 | -0.29 | -0.69 | -0.53 | 1.00 | 0.13 | -0.06 | -0.09 | 0.14 | 0.16 | 0.79 | 0.84 | -0.34 | 0.23 | -0.04 | 0.55 | 0.59 |
| | GUANINE | -0.62 | -0.69 | 0.48 | 0.48 | 0.48 | 0.19 | 0.27 | 0.13 | 1.00 | 0.94 | 0.92 | 0.99 | 0.96 | -0.28 | -0.13 | 0.35 | -0.67 | 0.65 | -0.05 | -0.34 |
| | HYPOXANTHINE | -0.67 | -0.69 | 0.57 | 0.57 | 0.57 | 0.35 | 0.40 | -0.06 | 0.94 | 1.00 | 0.98 | 0.95 | 0.92 | -0.38 | -0.26 | 0.55 | -0.73 | 0.68 | -0.12 | -0.46 |
| | URACIL | -0.68 | -0.67 | 0.57 | 0.57 | 0.57 | 0.37 | 0.40 | -0.09 | 0.92 | 0.98 | 1.00 | 0.93 | 0.91 | -0.40 | -0.29 | 0.52 | -0.69 | 0.72 | -0.16 | -0.49 |
| | XANTHINE | -0.63 | -0.68 | 0.47 | 0.47 | 0.47 | 0.19 | 0.28 | 0.14 | 0.99 | 0.95 | 0.93 | 1.00 | 0.97 | -0.24 | -0.09 | 0.38 | -0.68 | 0.64 | 0.01 | -0.32 |
| Other | 2,3-DIHYDROXYBENZOATE | -0.61 | -0.68 | 0.38 | 0.38 | 0.38 | 0.14 | 0.20 | 0.16 | 0.96 | 0.92 | 0.91 | 0.97 | 1.00 | -0.27 | -0.08 | 0.38 | -0.62 | 0.64 | -0.05 | -0.29 |
| | 4-GUANIDINOBUTANOATE | 0.25 | 0.13 | -0.59 | -0.59 | -0.59 | -0.66 | -0.58 | 0.79 | -0.28 | -0.38 | -0.40 | -0.24 | -0.27 | 1.00 | 0.80 | -0.41 | 0.54 | -0.46 | 0.56 | 0.77 |
| | GALACTITOL | 0.20 | 0.02 | -0.32 | -0.32 | -0.32 | -0.61 | -0.47 | 0.84 | -0.13 | -0.26 | -0.29 | -0.09 | -0.08 | 0.80 | 1.00 | -0.34 | 0.41 | -0.14 | 0.61 | 0.63 |
| | L-TRYPTOPHANAMIDE | -0.46 | -0.30 | 0.66 | 0.66 | 0.66 | 0.57 | 0.54 | -0.34 | 0.35 | 0.55 | 0.52 | 0.38 | 0.38 | -0.41 | -0.34 | 1.00 | -0.70 | 0.46 | -0.46 | -0.69 |
| | MYO-INOSITOL | 0.84 | 0.65 | -0.54 | -0.54 | -0.54 | -0.25 | -0.25 | 0.23 | -0.67 | -0.73 | -0.69 | -0.68 | -0.62 | 0.54 | 0.41 | -0.70 | 1.00 | -0.56 | 0.29 | 0.58 |
| | N-ACETYLMETHIONINE | -0.60 | -0.55 | 0.73 | 0.73 | 0.73 | 0.36 | 0.36 | -0.04 | 0.65 | 0.68 | 0.72 | 0.64 | 0.64 | -0.46 | -0.14 | 0.46 | -0.56 | 1.00 | -0.15 | -0.47 |
| | N-ACETYLNEURAMINATE | 0.26 | 0.10 | -0.43 | -0.43 | -0.43 | -0.47 | -0.32 | 0.55 | -0.05 | -0.12 | -0.16 | 0.01 | -0.05 | 0.56 | 0.61 | -0.46 | 0.29 | -0.15 | 1.00 | 0.73 |
| | S-ADENOSYLHOMOCYSTEINE | 0.40 | 0.22 | -0.78 | -0.78 | -0.78 | -0.67 | -0.71 | 0.59 | -0.34 | -0.46 | -0.49 | -0.32 | -0.29 | 0.77 | 0.63 | -0.69 | 0.58 | -0.47 | 0.73 | 1.00 |

Colour scale: 1.0, 0.5, 0, -0.5, -1.0

Matrix shows non-parametric Pearson correlation coefficient r values. Correlations were classed as non-significant (r -0.5–0.5; p > 0.05), weak (r = 0.5–06 or -0.5 to -0.6), moderate (r = 0.6–0.7 or -0.6 to -0.7), strong (r = 0.7–0.9 or -0.7 to -0.9), or very strong (r > 0.9 or < -0.9). All correlations of r = -0.5–0.5 had a p-value ≥ 0.05. The red colour denotes positive correlations while blue denotes negative correlations; the darker the colour, the stronger the correlation.

# Additional File Figure 4H – Correlation Matrix: PVC

| PVC | Amino Acids | | | Glucose Metabolism | | Purine Metabolism | | | | Other | |
|---|---|---|---|---|---|---|---|---|---|---|---|
| | N-ACETYLSERINE | PROLINE | SERINE | D-RIBOSE 5-PHOSPHATE | FRUCTOSE | DEOXYGUANOSINE | HYPOXANTHINE | INOSINE | URACIL | 2-METHYLMALEATE | N-ACETYLGLUTAMATE |
| **Amino Acids** | | | | | | | | | | | |
| N-ACETYLSERINE | 1.00 | 0.94 | 0.91 | -0.50 | -0.44 | -0.60 | 0.77 | -0.65 | 0.68 | -0.39 | 0.36 |
| PROLINE | 0.94 | 1.00 | 0.93 | -0.48 | -0.41 | -0.60 | 0.80 | -0.64 | 0.69 | -0.41 | 0.18 |
| SERINE | 0.91 | 0.93 | 1.00 | -0.38 | -0.42 | -0.55 | 0.77 | -0.62 | 0.62 | -0.42 | 0.30 |
| **Glucose Metabolism** | | | | | | | | | | | |
| D-RIBOSE 5-PHOSPHATE | -0.50 | -0.48 | -0.38 | 1.00 | 0.68 | 0.77 | -0.69 | 0.69 | -0.75 | 0.49 | -0.18 |
| FRUCTOSE | -0.44 | -0.41 | -0.42 | 0.68 | 1.00 | 0.78 | -0.70 | 0.74 | -0.67 | 0.78 | -0.29 |
| **Purine Metabolism** | | | | | | | | | | | |
| DEOXYGUANOSINE | -0.60 | -0.60 | -0.55 | 0.77 | 0.78 | 1.00 | -0.86 | 0.94 | -0.86 | 0.64 | -0.18 |
| HYPOXANTHINE | 0.77 | 0.80 | 0.77 | -0.69 | -0.70 | -0.86 | 1.00 | -0.87 | 0.95 | -0.68 | 0.32 |
| INOSINE | -0.65 | -0.64 | -0.62 | 0.69 | 0.74 | 0.94 | -0.87 | 1.00 | -0.88 | 0.74 | -0.09 |
| URACIL | 0.68 | 0.69 | 0.62 | -0.75 | -0.67 | -0.86 | 0.95 | -0.88 | 1.00 | -0.63 | 0.32 |
| **Other** | | | | | | | | | | | |
| 2-METHYLMALEATE | -0.39 | -0.41 | -0.42 | 0.49 | 0.78 | 0.64 | -0.68 | 0.74 | -0.63 | 1.00 | -0.02 |
| N-ACETYLGLUTAMATE | 0.36 | 0.18 | 0.30 | -0.18 | -0.29 | -0.18 | 0.32 | -0.09 | 0.32 | -0.02 | 1.00 |

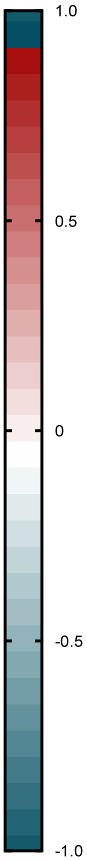


Matrix shows non-parametric Pearson correlation coefficient r values. Correlations were classed as non-significant (r -0.5–0.5; p > 0.05), weak (r = 0.5–06 or -0.5 to -0.6), moderate (r = 0.6–0.7 or -0.6 to -0.7), strong (r = 0.7–0.9 or -0.7 to -0.9), or very strong (r > 0.9 or < -0.9). All correlations of r = -0.5–0.5 had a p-value ≥ 0.05. The red colour denotes positive correlations while blue denotes negative correlations; the darker the colour, the stronger the correlation.
